# CircGPRC5A enhances colorectal cancer progress by stabilizing PPP1CA and inducing YAP dephosphorylation

**DOI:** 10.1186/s13046-023-02915-7

**Published:** 2023-12-06

**Authors:** Zhenzhou Chen, Yidan Li, Kuan He, Jianguo Yang, Qican Deng, Yajun Chen, Zhongxue Fu

**Affiliations:** 1 Department of Gastrointestinal Surgery, The Third Affiliated Hospital of Chongqing Medical University, Chongqing, China; 2 Department of Cardiology, The Second Affiliated Hospital of Chongqing Medical University, Chongqing, China; 3 Department of Gastrointestinal Surgery, The First Affiliated Hospital of Chongqing Medical University, Chongqing, China

**Keywords:** circGPRC5A, Colorectal cancer, PPP1CA, UBA1, YAP

## Abstract

**Background:**

With the advancements in bioinformatic technology, an increasing number of circular RNAs (circRNAs) have been discovered and their crucial roles in the development and progression of various malignancies have been confirmed through multiple pathways. However, the specific mechanisms involving protein-binding circRNAs in colorectal cancer (CRC) remain largely unexplored.

**Methods:**

Differential circRNA expression was assessed using a human circRNA microarray in five CRC tissue and paired normal samples. CircGPRC5A expression was then confirmed in the CRC tissues and paired normal samples using qRT-PCR. The biological function of circGPRC5A in CRC were studied in vitro and in vivo. Western blotting, fluorescence in situ hybridization, immunofluorescence, RNA pulldown, mass spectrometry, immunoprecipitation, quantitative phosphoproteomics, and RNA-binding protein immunoprecipitation assays were used to study circGPRC5A.

**Results:**

Our analysis revealed that circGPRC5A expression was higher in CRC tissues compared to normal tissues and was associated with tumor size, tumor stage and lymph node status. CircGPRC5A promoted CRC cell proliferation, migration, and metastasis in vitro and in vivo. CircGPRC5A could stabilize PPP1CA protein by inhibiting the binding between UBA1 and PPP1CA, and increasing YAP dephosphorylation.

**Conclusions:**

Our study revealed that circGPRC5A plays an essential function in CRC progression by stabilizing PPP1CA protein and enhancing YAP dephosphorylation. CircGPRC5A could act as a novel and potential target for CRC.

**Supplementary Information:**

The online version contains supplementary material available at 10.1186/s13046-023-02915-7.

## Introduction

Colorectal cancer (CRC) ranks as the third most common malignancy of the digestive system and is the second major cause of cancer mortality [1, 2]. Despite advancements in screening programs and therapeutic approaches, the 5-year survival rate remains alarmingly low (< 12%), especially for CRC patients with metastasis or recurrence [3–5]. This highlights the pressing need for novel and effective molecular targets.

Circular RNAs (circRNAs) lack a 5′-cap or 3′-polyadenylation tail, making them more stable and resistant to exonucleases compared with linear RNAs [6, 7]. Bioinformatics studies show that circRNA dysregulation is tied to cancer progression [8]. Although circRNAs were initially considered functionally inert, circRNAs acting as microRNA (miRNA) sponges are frequently reported [9]. In addition, circRNAs may have additional properties such as interacting with proteins in multiple signaling pathways of cancer [10]. Unlike circRNA-miRNA interactions, protein-binding circRNA interactions regulate protein expression, degradation, and biogenesis to promote cancer development [11–13]. However, the consequence of protein-binding circRNA interactions in CRC is still largely unknown.

Protein Phosphatase 1 Catalytic Subunit Alpha (PPP1CA) encodes PP1A, which is one of the three catalytic subunits of protein phosphatase 1 (PP1) that is specific for serine/threonine [14, 15]. Protein kinases and phosphatases regulate protein phosphorylation, which influences many physiological processes. The imbalance of PPP1CA leads to the development of multiple diseases. For example, PPP1CA can dephosphorylate the Ser418 residue of FOXP3 in regulatory T-cells (Treg), thereby rendering Treg cells functionally defective in rheumatoid arthritis patients [16]. For cancer patients, PPP1CA is often overexpressed, and some studies have linked expression with clinicopathological characteristics [17, 18]. However, PPP1CA’s role in cancer development, notably in CRC, is unknown.

The Hippo signaling pathway protein Yes-associated protein (YAP) is crucial to many biological processes. [19–21]. Ser127 phosphorylation can result in YAP’s interaction with 14–3-3 proteins, inducing cytoplasmic retention of YAP. YAP Ser381 phosphorylation, on the other hand, can trigger ubiquitination and degradation of YAP [22]. However, dephosphorylation of YAP may cause translocation from the cytoplasm to nucleus to interact with TEAD1–4 transcription factors (TEADs), triggering target gene expression and cell proliferation and apoptosis [23, 24]. Therefore, the imbalance of YAP phosphorylation can lead to the occurrence and development of numerous malignancies.

In our study, we focused on a circRNA from the *GPRC5A* gene (has_circRNA_101017 (circBase ID: has_circ_0025506)), circGPRC5A, using a human circRNA array to profile circRNAs expression in CRC. CRC tissue was found to have higher levels of circGPRC5A than adjacent normal tissues. CircGPRC5A promoted CRC progression by competitively inhibiting the binding of UBA1 and PPP1CA, thereby preventing PPP1CA ubiquitination. This caused a low phosphorylation state of YAP, and ultimately could lead to CRC development. Our results suggest that circGPRC5A can contribute to improving the understanding of the roles of circRNA in CRC and can act as a novel and potential therapeutic target for CRC patients.

## Materials and methods

### Patients and clinical samples

Between October 2021 and March 2022, 80 pairs of human CRC samples and paired adjacent non-tumor tissues were collected from the First Affiliated Hospital of Chongqing Medical University. All samples came from CRC patients who underwent surgical resections, and they were immediately frozen in liquid nitrogen before being moved to − 80 °C for long-term storage. The clinicopathological data of all CRC patients were gathered and examined from electronic medical records. All patients gave their informed consent, which was evaluated and approved by the First Affiliated Hospital of Chongqing Medical University’s ethics committee (NO.2020–358).

### Cell culture and transfection

The human CRC cell lines HT29, HCT116, HCT8 and SW480 were purchased from Cell Bank of the Chinese Academy of Sciences (Shanghai, China). The normal colonic epithelial cell line NCM460 was obtained from the American Type Culture Collection (Manassas, VA, USA). HT29, HCT116, HCT8, SW480 and NCM460 were cultured in Dulbecco’s Modified Eagle medium (DMEM) supplemented with 10% fetal bovine serum (BI, Israel) and 1% penicillin/streptomycin (Gibco, USA). Cells were grown in an incubator at 37 °C and 5% CO_2_.

SiRNA and plasmids were transfected into CRC cell lines using Lipofectamine 3000 reagent in accordance with the manufacturer’s instructions (Invitrogen, CA, USA). Empty vector (EV) and negative control siRNA were used as controls. The siRNA sequences targeting circGPRC5A and UBA1 are shown in Table S1. Lentivirus-mediated short hairpin RNA (shRNA targeting circGPRC5A and PPP1CA), lentiviruses overexpressing circGPRC5A and control lentivirus were purchased from Han Biotechnology (Shanghai, China). After lentiviral transduction of CRC cells to generate stable knockdowns of circGPRC5A and PPP1CA and stable overexpression of circGPRC5A, cells were screened with puromycin (2 mg/ml).

### Human circRNA microarray

Five CRC tissues and paired para-cancerous tissues were compared using a human circRNA microarray. Thermo Fisher Scientific’s NanoDrop 1000 was used to measure the purity as well as the concentration of the total RNA that was extracted using TRIzol (Invitrogen, CA, USA). Rnase R (Thermo Fisher Scientific, Waltham, USA) was utilized to process total RNA from each sample to enrich for circular RNA, which was then amplified and turned into fluorescent circRNA using random primers in accordance with the Arraystar Super RNA Labeling methodology. Using an Agilent Hybridization Oven (Agilent Technologies, CA, USA), the tagged circRNAs were hybridized onto the Arraystar Human CircRNA Arrays V2 (8 × 15 K, Arraystar, Rockville, USA) and cultured for 17 h at 65 °C. After washing, slides were examined using the Agilent Scanner G2505C (Agilent Technologies, CA, USA). Finally, data were collected and analyzed using Agilent Feature Extraction software and the R software limma package.

### RNA extraction and qRT-PCR

Total RNA was isolated from CRC tissues and cells using TRIzol reagent (Invitrogen, CA, USA). The Cytoplasmic and Nuclear RNA Purification Kit (Norgen Biotek, Canada) was used to extracted nuclear and cytoplasmic RNA from CRC cells according to the manufacturer’s guidelines. The concentration and purity of total RNA was determined using the NanoDrop 2000 (Thermo Fisher Scientific, MA, USA). cDNA was generate using the RT Reagent Kit (Takara, Japan) and the TB Green Kit (Takara, Japan) was used for qRT-PCR. The 2^-ΔΔCt method was used to calculate the relative expression level of circRNA and mRNA, and was normalized to *GAPDH*. Primer sequences can be found in Table S2.

### Cell Counting Kit-8 and colony formation assays

Transfected CRC cells were inoculated into a 96-well plate at a density of 1000 cells per well. A total of 10 uL of the Cell Counting Kit-8 (CCK-8) solution (APExBIO, USA) was used to measure cell proliferation at 0, 24, 48, 72, and 96 h after seeding. The plate was cultured for 2 h at 37 °C in 5% CO_2_, followed by measurement of the absorbance at 450 nm. For colony formation assays, 200 or 1000 transfected CRC cells were seeded into 24-well plates or 6-well plates and cultured for 7 days at 37 °C in 5% CO_2_. When colonies formed, the plates were preserved with 4% paraformaldehyde and stained with 0.1% crystal violet.

### 5-ethynyl-2′-deoxyuridine assay

The cell proliferation was measured using a 5-ethynyl-2′-deoxyuridine (EdU) Cell Proliferation Kit (Beyotime, China). Briefly, transfected CRC cells were seeded into 96-well plates and incubated for 24 h at 37 °C in 5% CO_2_. Next, 50 µl/L EdU solution was added to each well, and the plates were cultured at 37 °C in 5% CO_2_ for 2 h. After three washes in PBS, the cells were then fixed for 30 min at room temperature with 4% paraformaldehyde.

Triton X-100 (1%) was used to permeabilize the transfected CRC cells. The cells were then stained with Azide 594 and Hoechst 33,342. Confocal laser scanning microscopy was used to image the cells.

### Transwell and wound healing assays

For the Transwell migration assay, transfected CRC cells (5 × 10^4^) were resuspended in 200 µL serum-free DMEM and plated in the upper chamber (Corning, USA), while the lower chamber included DMEM with 15% fetal bovine serum. After 48 h, 4% paraformaldehyde was used to fix the invaded cells and 1% crystal violet was used to stain these cells. Matrigel (BD, USA) was used to coat the upper chamber in the Transwell invasion assay, and cells were treated as previously described.

To assess the migratory ability of the tumor cells, a wound healing assay was used. Transfected CRC cells were plated into 6-well plates and incubated until the cell density was at least 90%. The bottom of the culture plates was scratched with a sterile pipette tip to make wounds. After washing off the detached cells with PBS, serum-free DMEM was added to the cells for 24 h. Finally, the scratch was examined and imaged at 0 and 24 h to analyze the number of migrated cells.

### RNase R treatment

RNase R (Geneseed Technologies, China) was used to purify circRNA. Total RNA (4 µg) was mixed with 10 × reaction buffer (2 µL) and RNase R (3–4 U/µg RNA). Then the reaction was brought to 20 µL using RNase-free Water. The sample was incubated for 10 min at 37 °C. Finally, qRT-PCR was performed to assess the relative expression levels of circRNA and mRNA.

### Actinomycin D assay

For the actinomycin D assay, 2 mg/mL Actinomycin (Sigma-Aldrich, USA) was used to block gene transcription and to assess the half-life of circRNA. Cells were harvested at 0, 4, 8, and 24 h and the relative expression levels of circRNA and mRNA were assessed using qRT-PCR.

### Western blot analysis

RIPA Lysis Buffer (Beyotime, China), which includes 1% PMSF and 1% phosphatase inhibitor, was used to lyse cells. The BCA Protein Assay Kit (Beyotime, China) was used to measure total protein concentrations. Identical amounts of protein were separated on an 8% or 10% SDS-PAGE gel and then transferred onto 0.22 um polyvinylidene fluoride membranes (PVDF, Millipore, Germany). The PVDF membranes were blocked with 5% skim milk or bovine serum albumin for 1.5 h and then incubated with specific antibodies for 12–16 h at 4 °C. The primary antibodies used were PPP1CA (sc-7482, Santa Cruz Biotechnology), Ub (#3936S CST), UBA1 (ab180125, Abcam), FBXO2 (ab133717, Abcam), YAP-Ser127 (AP0489, ABclonal), YAP-Ser109 (YP1553, Immunomy), YAP-Ser61(#75784S, CST), YAP (A21216, ABclonal), Pan Phospho-Serine/Threonine (T91067S, Abmart), and GAPDH (10,494–1-AP, Proteintech). The next day, the PVDF membranes were treated with HRP-conjugated goat anti-rabbit or anti-mouse IgG for 2 h. Membranes were washed with TBST 3X, and analysis was performed using the ECL detection system.

### Fluorescence in situ hybridization and immunofluorescence

A Cy3-labeled circGPRC5A probe (Sangon, China) was used for fluorescence in situ hybridization (FISH) (Table S3). RNA-FISH was performed using the FISH Kit (Ribobio, China) to visualize the localization and expression of circGPRC5A in CRC cells. According to the manufacturer’s protocol, Cy3-labeled circGPRC5A probes were cultured with CRC cells for 16 h at 37 °C. CRC cells were then washed in different concentrations of saline sodium citrate buffer solution. DAPI was used to stain the nuclei of CRC cell after washing CRC cells. To further assess the colocalization of circGPRC5A and PPP1CA, CRC cells were transfected with Cy3-labeled circGPRC5A probes and cultured with anti-PPP1CA antibody. Finally, laser confocal microscopy was used to image the cells.

### CircGPRC5A pulldown assay

A circRNA pulldown assay with MS2-capturing protein was used to evaluate the RNA-binding proteins associated with circGPRC5A (Geneseed, China). Briefly, circGPRC5A-MS2 and MS2-CP-Flag vectors were transfected into HCT116 cells. Then, MS2-CP-Flag antibodies were combined with the capture protein MS2-CP-Flag to further enrich for circRNA pulldown products. Finally, circGPRC5A pulldown products and the negative control were analyzed using mass spectrometry (Q Exactive, Thermo Scientific, USA).

### RNA-binding protein immunoprecipitation assay

The RNA immunoprecipitation (RIP) assay was conducted using a Magna RIP RNA-Binding Protein Immunoprecipitation Kit (Millipore Magna, USA) to explore the relationship between circGPRC5A and PPP1CA. In brief, CRC cells were lysed and collected using RIP lysis buffer. Target antibody and lysate were mixed and agitated for 6 h at 4 °C. After washing, TRIzol reagent was used to extract the immunoprecipitated (IPed) and enriched circRNA. The enriched circRNA was then subjected to qRT-PCR analysis.

### Co-immunoprecipitation assay

Co-immunoprecipitation (co-IP) was conducted using a Co-IP Kit (Thermo Scientific, USA). Briefly, CRC cell lysates were incubated with target antibody for 12 h at 4 °C. The mixture, including antigen and target antibody, were combined with protein A/G magnetic beads for 2 h at room temperature. Next, the A/G magnetic bead and lysate mixture was washed with IP lysis/rinsing buffer. Finally, antigen–antibody complexes were eluted for 10 min at 95 °C. The co-IP products were analyzed using mass spectrometry (Q Exactive, Thermo Scientific, USA) and western blotting.

### 4D label-free quantitative phosphoproteomics analysis

Phosphoproteomics was used to analyze the changes of protein phosphorylation levels in the transfected CRC cells. In brief, protein was isolated from the transfected CRC cells and the protein concentration was evaluated using the BCA or Bradford assays. Then, SDS-PAGE electrophoresis analysis was performed with the protein samples to evaluate whether the sample quality met the requirements for subsequent experiments. High-quality sample was reduced and alkylated, and equal amount of each sample were taken for trypsin hydrolysis and liquid chromatography coupled with tandem mass spectrometry analysis. Finally, data statistics and bioinformatics analyses were conducting according to search results obtained from the MaxQuant Software.

### Immunohistochemistry

Tissue samples were fixed with 4% formaldehyde. After embedding in paraffin, tissue blocks were cut into 4um sections. After dewaxing and hydrating, a 3% hydrogen peroxide solution was added to the tissue sections to inhibit endogenous peroxidase activity. Anti-PPP1CA and anti-Ki 67 antibody solutions were added to the sections and incubated on the tissues for 12–16 h at 4 °C with anti-PPP1CA and anti-Ki 67 antibodies. Brown precipitates were produced after 3,3'-diaminobenzidine incubation. Finally, the sections were counter-stained using hematoxylin. Immunohistochemistry (IHC) images were obtained using an Olympus microscope.

### Animal studies

The Animal Care Committee of Chongqing Medical University reviewed and approved the animal experimental protocol (NO. IACUC-CQMU-2022–0019). For xenografts in mice, 4–6-week-old female BALB/c nude mice were purchased from Chongqing Ensiweier Biotechnology Ltd. Next, 1 × 10^7^ cells were injected into the right subcutaneous axilla (*n* = 5 per group). The long and short axes of the subcutaneous tumors were measured on days 5, 10, 15, 20, and 25 after injection, and the formula V = (width^2^ + long)/2 was used to calculate the subcutaneous tumor volume. Mice were euthanized 25 days after tumor injection. The subcutaneous tumors were isolated from the nude mice, the tumors were weighed, and the mass and volumes were measured. Tumors were fixed in a 4% formalin solution for hematoxylin and eosin staining and IHC.

We constructed a liver metastasis model to explore the metastatic ability of cancer cells. A total of 3 × 10^6^ cells were injected into the spleens of nude mice. Mice were sacrificed after 25 days. Liver tissue sections were stained with hematoxylin and eosin, and tumor colonies were examined under a microscope.

### Statistical analysis

All experiments were independently repeated three times and the data are presented as mean ± standard deviation (SD). The Chi-square test or Fisher’s precision probability test was used to analyze the relationship between circGPRC5A and CRC patient clinical characteristics. A Wilcoxon signed-rank analysis was conducted to test non-normal distribution paired samples. A Mann–Whitney test was applied to analyze the data with non-matched and non-normally distributed samples. The comparison of two samples was assessed using a Student’s t-test, and the comparison of three or more samples was performed using a one-way ANOVA analysis. Correlation analyses were conducted using a Spearman’s rank correlation coefficient test. *P* < 0.05 was considered statistically significant. The statistical analysis was performed using R software (V4.0.5) and GraphPad Prism 8.

## Results

### Identification and characterization of circGPRC5A in CRC using a microarray analysis

**Fig. 1:**
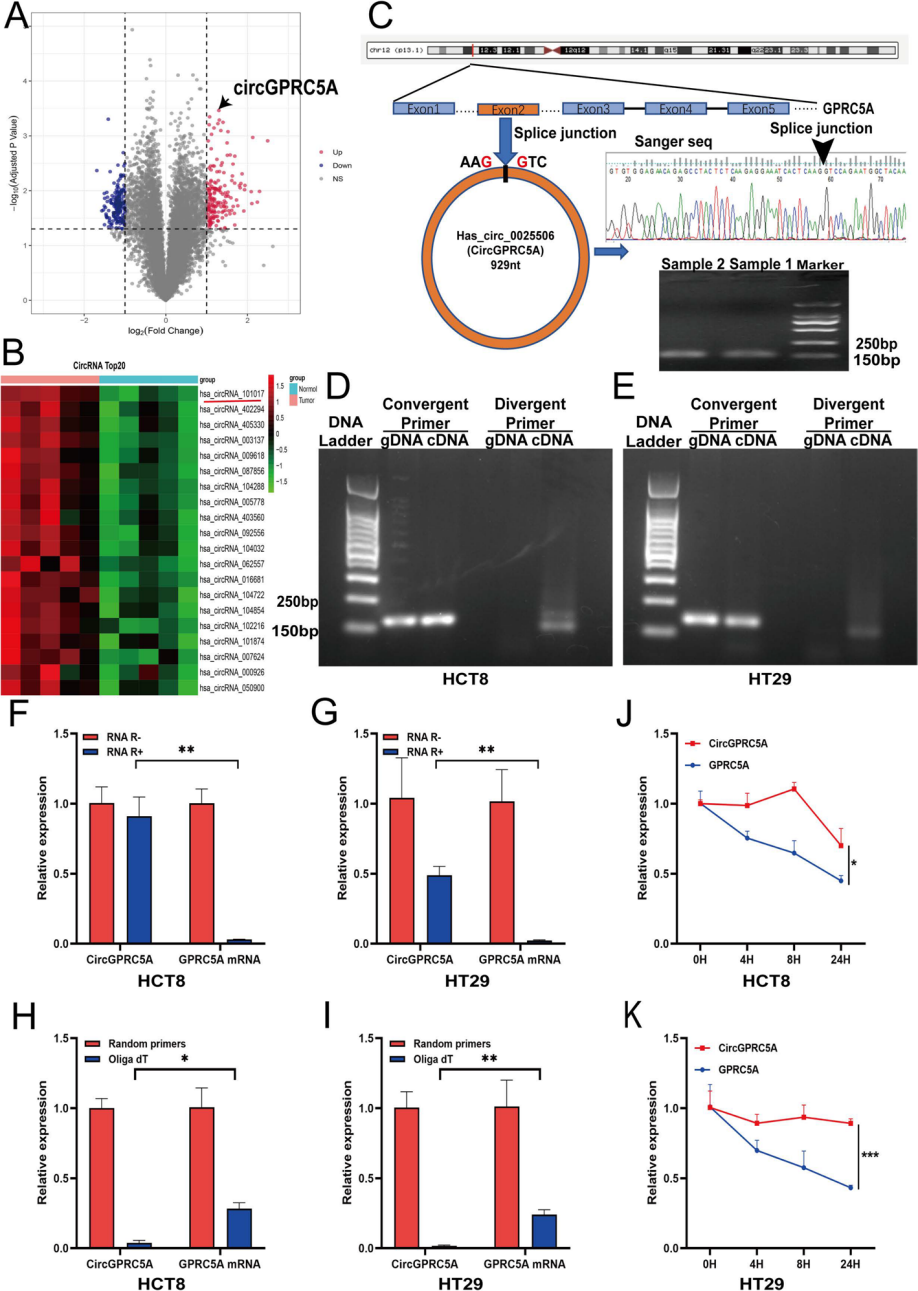
Human circRNA microarray analysis was used to characterize circGPRC5A in CRC. **A** Volcano plot of human circRNA microarray demonstrating upregulated or downregulated circRNAs in five CRC and paired normal tissues. **B** Human circRNA microarray cluster heat map displaying the top 20 upregulated circRNAs in five CRC and paired normal tissues. **C** Northern blotting and Sanger sequencing were used to characterize circGPRC5A. **D**–**E** CircGPRC5A was amplified from the CRC cell line cDNA (HCT8 and HT29), but not from gDNA, using divergent primers. **F**–**G** After RNase R treatment, qRT-PCR was used to confirm the expressions of circGPRC5A and GPRC5A mRNA in HCT8 and HT29 cells. **H**–**I** Using oligo dT and random 6-mers, we investigated the levels of circGPRC5A and GPRC5A mRNA expressions in HCT8 and HT29 cells. **J**–**K** The expressions of circGPRC5A and GPRC5A mRNA were assessed using qRT-PCR in HCT8 and HT29 cells following treatment with actinomycin D at the appropriate timepoint. The results of each assay, which were carried out three times, are displayed as the mean ± SD. *P < 0.05; **P < 0.01; ***P < 0.001

Using a circRNA microarray, we identified 596 upregulated and 453 downregulated circRNAs in five CRC tissues compared with adjacent normal tissues (Fig. 1A). A clustered heat map revealed the differentially expressed top 20 circRNAs (Fig. 1B). Among these circRNAs, hsa_circ_0025506 (circGPRC5A) showed the greatest differential upregulation. CircGPRC5A is derived from exon 2 of *GPRC5A* and is located on chr12:13,061,176–13062105. To verify the closed-loop structure of circGPRC5A, we confirmed the presence of a splice junction in the PCR product of circGPRC5A by using Sanger sequencing (Fig. 1C). To further confirm the characteristics of circGPRC5A, we amplified circGPRC5A using divergent primers from cDNA, and not from gDNA, of CRC cell lines (HCT8 and HT29) (Fig. 1D, E). RNase R experiments demonstrated that the level of GPRC5A mRNA was reduced compared to levels of circGPRC5A (Fig. 1F, G). In addition, qRT-PCR demonstrated that the relative circGPRC5A levels were significantly higher in random hexamers than in oligo dT 18 primers (Fig. 1H, I). On the other hand, the relative circGPRC5 RNA levels were more stable than GPRC5A mRNA levels after actinomycin D treatment of HCT8 and HT29 cells (Fig. 1J, K). In conclusion, these findings indicate that circGPRC5A has a closed-loop structure.

### CircGPRC5A is upregulated in CRC and is correlated with clinicopathologic features

**Fig. 2:**
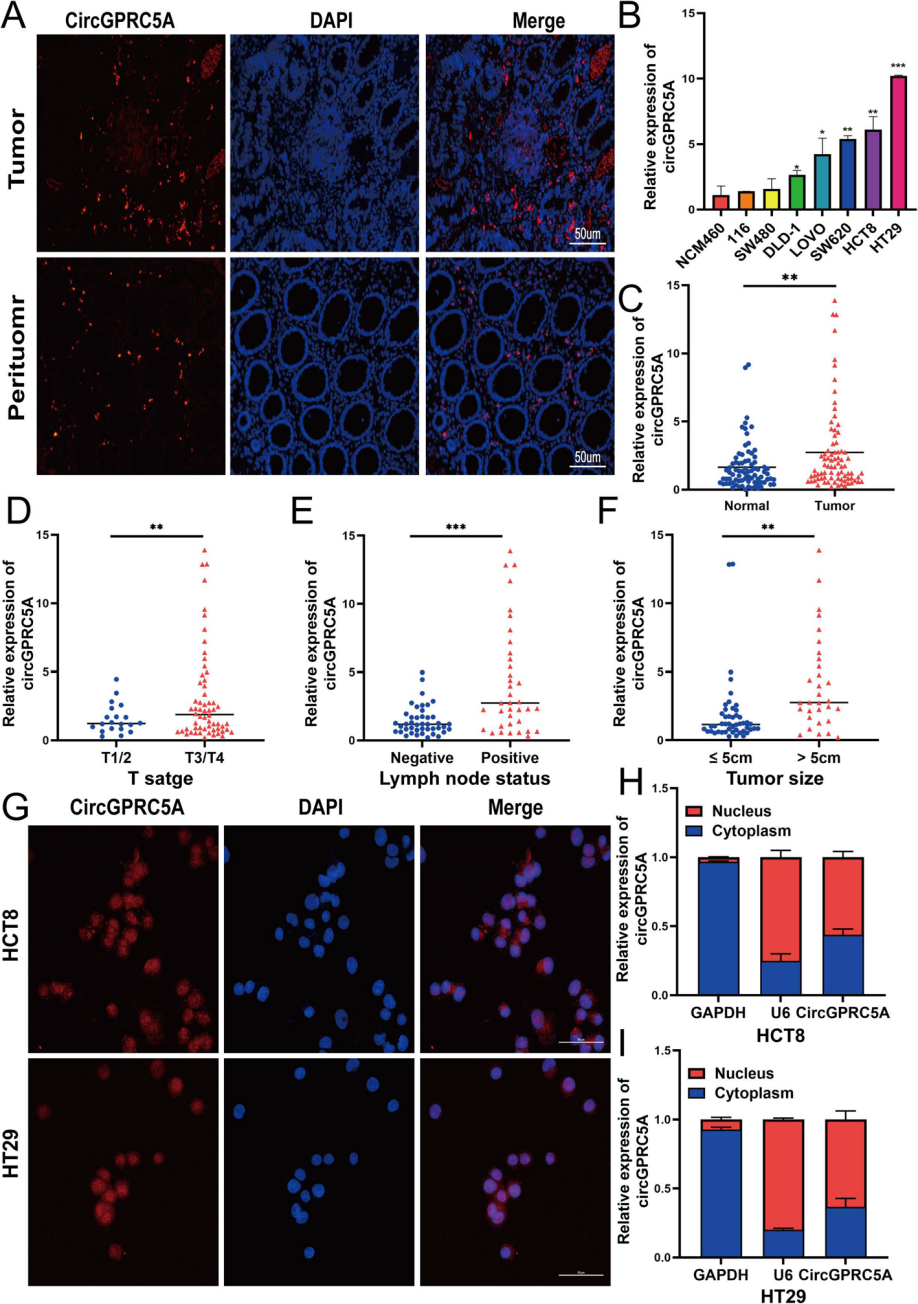
CircGPRC5A expression in CRC cell lines and correlations between circGPRC5A expression and CRC patients with clinicopathologic features. **A** FISH was used to investigate the expression of circGPRC5A in paired normal tissues and CRC tissues. **B** The qRT-PCR was used to confirm the expressions of circGPRC5A in NCM460 and CRC cell lines. **C** CircGPRC5A expressions in matched normal tissues and 80 CRC tissues. **D**-**F** T stage, lymph node status and Tumor size were considered when evaluating the expression of circGPRC5A. **G** The cellular localizations of circGPRC5A in HCT8 and HT29 cells were investigated using FISH. **H**-**I** The qRT-PCR was used to assess the expressions of circGPRC5A in nuclear and cytoplasmic RNA of HCT8 and HT29 cells. Based on data from three independent experiments, values are presented as the mean ± SD. *P < 0.05; **P < 0.01; ***P < 0.001

Three CRC tissues and paired para-cancerous tissues were compared using the circGPRC5A FISH probe, which revealed that the expression of circGPRC5A in CRC tissue was higher than in matched adjacent normal tissues (Fig. 2A, Figure S1A–C). In addition, circGPRC5A was significantly upregulated in DLD-1, LOVO, SW620, HCT8 and HT29 cells compared with NCM460 cells (Fig. 2B). Extracted RNA from the 80 CRC tissues and corresponding adjacent normal tissues were subjected to qRT-PCR analysis to further confirm the presence of circGPRC5A in CRC tissues. CircGPRC5A was significantly upregulated in CRC tissues when compared to nearby normal tissues, which is consistent with our circRNA microarray data from CRC tissues (Fig. 2C).

Furthermore, high circGPRC5A tissue expression had a strong correlation with tumor size, lymph node status, T stage, and TNM stage (Fig. 2D–F) (Table S4). The distribution of circGPRC5A in HT29 and HCT8 cells was investigated using nuclear-cytoplasmic fractionation and FISH. In HCT8 and HT29 cells, the nuclear localization of circGPRC5A was slightly higher compared with cytoplasmic localization (Fig. 2G–I).

### CircGPRC5A promotes CRC cell proliferation, migration and invasion in vitro

**Fig. 3:**
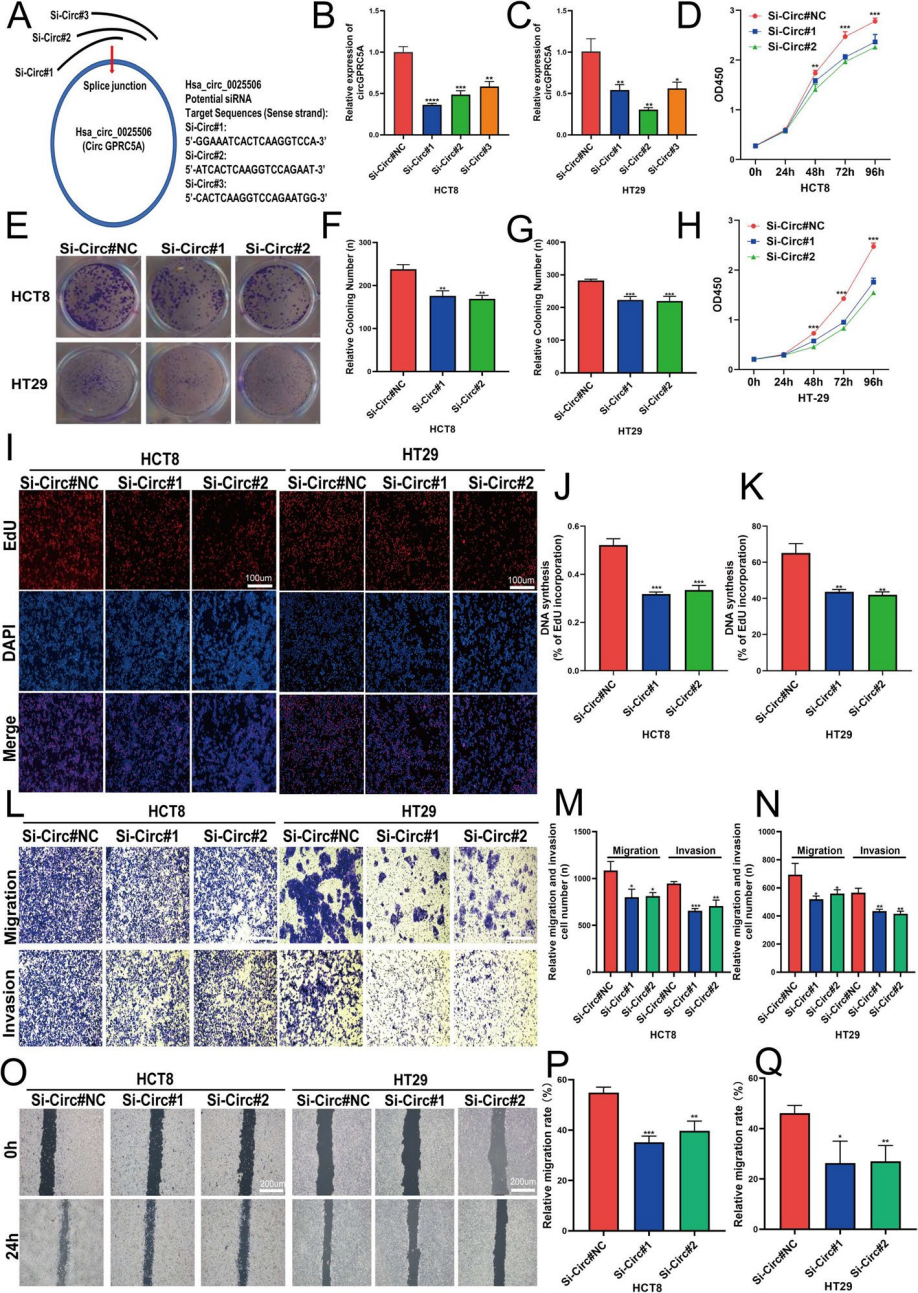
CircGPRC5A promotes CRC cell invasion, migration, and proliferation in vitro. **A** Schematic illustration of the siRNA targeted at the back-splice junctions of circGPRC5A. **B**–**C** After transfecting HCT8 and HT29 cells with siRNA or siNC, circGPRC5A expressions were assessed using qRT-PCR. **D**–**H** Colony formation and CCK-8 assays were used to measure proliferation after siRNA or siNC transfection into HCT8 and HT29 cells. **I**–**K** EdU assay (scalebar: 100 µm) was used to determine whether HCT8 and HT29 cells could proliferate after circGPRC5A silencing. **L**–**N** Transwell assays were performed to assess HCT8 and HT29 cell migration and invasion abilities after circGPRC5A silencing. **O**–**Q** Following circGPRC5A silencing, wound healing assays were carried out to measure migration and invasion of HCT8 and HT29 cells. Based on data from three independent experiments, values are presented as the mean ± SD. *P < 0.05; **P < 0.01; ***P < 0.001

Tumor development and progression are significantly influenced by cell maintenance processes like proliferation and apoptosis resistance [25]. The expression of circGPRC5A was higher in HT29 and HCT8 cells compared with other CRC cell lines. Therefore, HT29 and HCT8 cells were used in subsequent studies. HT29 and HCT8 cells were transfected with si-circGPRC5A#1, si-circGPRC5A#2, si-circGPRC5A#3 and si-circGPRC5A#NC. qRT-PCR revealed that si-circGPRC5A#1 and si-circGPRC5A#2 gave a better knockdown efficiency compared with si-circGPRC5A#3 (Fig. 3A–C). We therefore used si-circGPRC5A#1 and si-circGPRC5A#2 to investigate the functional effects of circGPRC5A silencing in HT29 and HCT8 cells. CCK-8, colony formation, and EdU-incorporation revealed that silencing circGPRC5A reduced proliferation and colony formation (Fig. 3D–K).

**Fig. 4:**
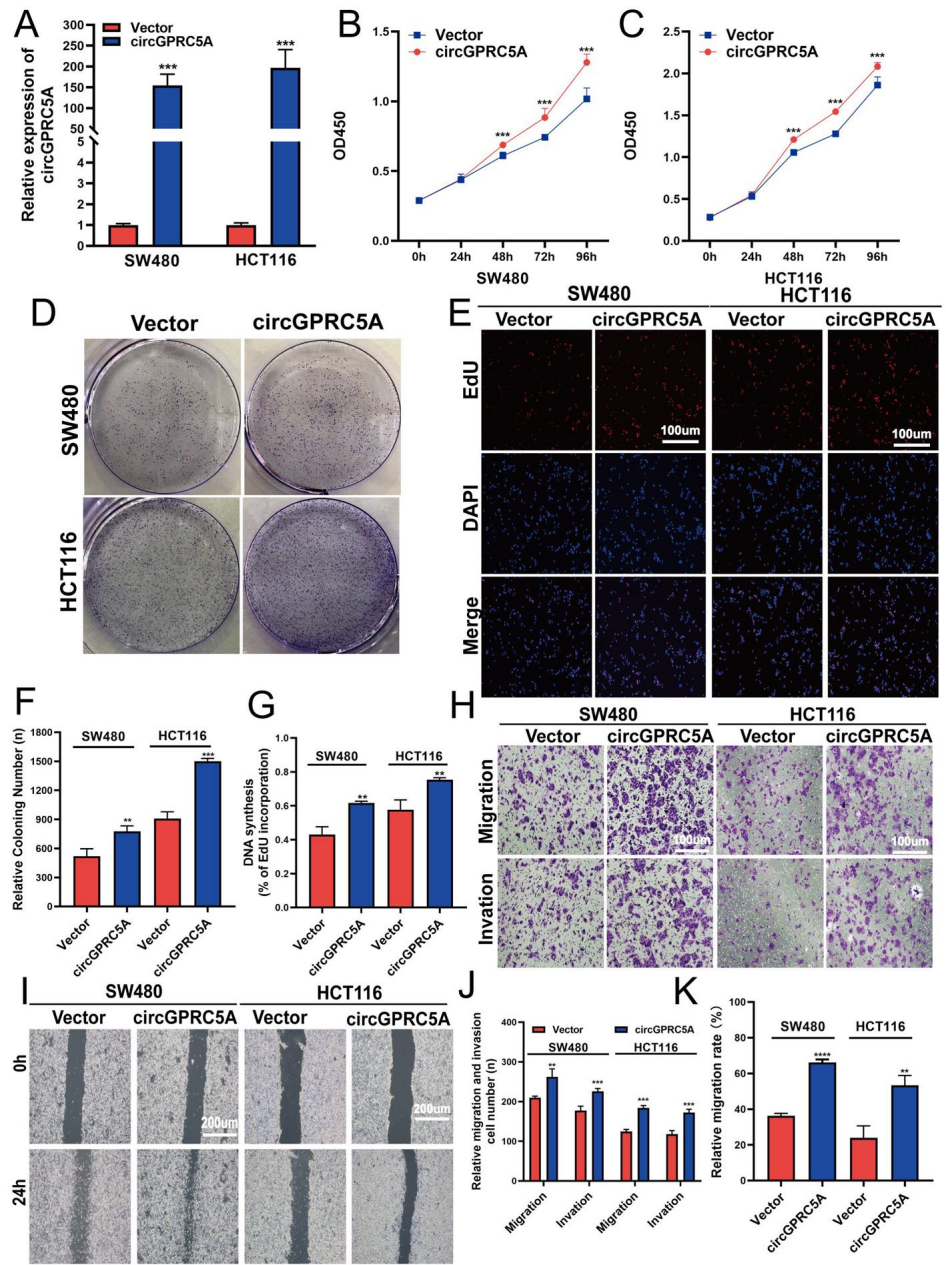
CircGPRC5A promotes CRC cell invasion, migration, and proliferation in vitro. **A** CircGPRC5A overexpressions in HCT116 and SW480 were confirmed using qRT-PCR. **B**–**G** Following circGPRC5A overexpressions in SW480 and HCT116 cells, CCK-8, colony formation, and Edu assays were used to assess proliferation. **H**–**K** Transwell and wound healing assays were used to measure migration and invasion following circGPRC5A overexpressions in SW480 and HCT116 cells. Based on data from three independent experiments, values are presented as the mean ± SD. *P < 0.05; **P < 0.01; ***P < 0.001. Following transfection of MS2-CP and circGPRC5A-MS2 into CRC cells, fluorescence and bright field images were obtained

Previous results indicate that circGPRC5A expression is positively correlated with CRC patient lymph node status and TNM stage. Hence, to ascertain how circGPRC5A affects the migration and invasion of CRC cells, Transwell and wound healing assays were carried out. CircGPRC5A silencing significantly suppressed the migration and invasion abilities of HT29 and HCT8 cells (Fig. 3L–Q). We further explored the potential functional role of circGPRC5A by stably overexpressing circGPRC5A in CRC cell lines with lower circGPRC5A, HCT116 and SW480 cells, using lentiviral infection (Fig. 4A). Functionally, circGPRC5A overexpression significantly improved colony formation and cell proliferation as assessed by the CCK-8, colony formation, and EdU assays (Fig. 4B–G). The migration and invasion capacities of HCT116 and SW480 cells were also markedly enhanced with circGPRC5A overexpression (Fig. 4H–K). These data support that circGPRC5A can function as a tumor promotor to contribute to CRC development.

### CircGPRC5A interacts with PPP1CA and stabilizes PPP1CA protein

**Fig. 5:**
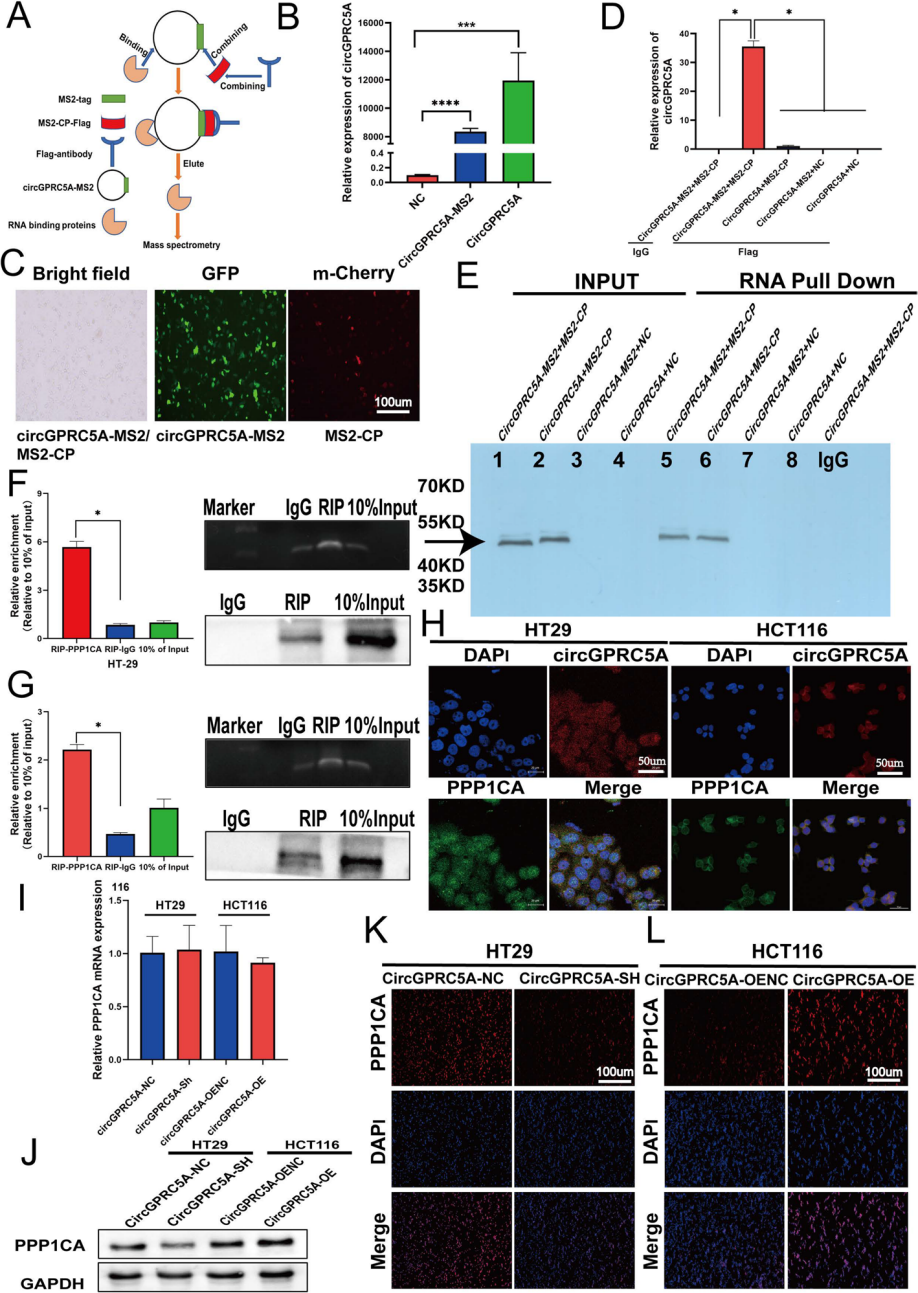
PPP1CA protein may be stabilized by circGPRC5A-PPP1CA binding. **A** RNA pulldown using the MS2-tagging system is outlined in a flow chart. **B** Following transfection of the expression plasmids circGPRC5A, NC, and circGPRC5A-MS2 into CRC cells, the relative expression of circGPRC5A was assessed using qRT-PCR. **C** Following transfections of MS2-CP and circGPRC5A-MS2 into CRC cells, fluorescence and bright field images were obtained. **D** The qRT-PCR was used to measure the enrichment of circGPRC5A in a complex with MS2-CP-Flag **E** MS2 combined protein in various groups in the RNA pulldown assay was examined using western blotting. **F**-**G** CircGPRC5A and PPP1CA were compared using the RIP assay to assess their relationship. The qRT-PCR was used to identify circGPRC5A expressions in the immunoprecipitates. **H** Immunofluorescence-FISH was used to examine co-localizations of circGPRC5A and PPP1CA in HT29 and HCT116 cells. **I** Following the overexpression or silencing of circGPRC5A, the relative expression of PPP1CA mRNA was assessed using qRT-PCR. **J**–**L** PPP1CA protein levels were assessed using western blotting and immunofluorescence after circGPRC5A was silenced or overexpressed. Values from three independent experiments are presented as the mean ± SD. *P < 0.05; **P < 0.01; ***P < 0.001

CircGPRC5A can promote bladder oncogenesis and metastasis by circGPRC5A-targeting peptide, and promote liver cancer progression by binding microRNAs [26, 27]. However, the mechanisms of circGPRC5A protein-binding are still unknown. Since circGPRC5A was distributed in both the cytoplasm and nucleus, we investigated protein-binding circRNA complexes influenced by circGPRC5A in CRC. First, we conducted proteomic profiling by using a circRNA pulldown assay with MS2-CP-Flag (Fig. 5A). CircGPRC5A-MS2 vector and MS2-CP-Flag were transfected into HCT116 cells, and qRT-PCR was used to verify the transfection efficiency (Fig. 5B,C). Then, a MS2-CP-Flag antibody was mixed with capture protein MS2-CP-Flag to further enrich for circRNA pulldown products. In the capture eluate, qRT-PCR revealed that circGPRC5A was highly abundant (Fig. 5D, E). Both assays successfully demonstrated the circRNA pulldown assay specificity. After mass spectrometry analysis of circRNA pulldown products, 33 candidate circGPRC5A-binding proteins were identified by comparing the circGPRC5A + MS2-CP-tansfected group with the circGPRC5A-MS2 + MS2-CP-transfected group. Mass spectrometry revealed that the value of protein matches and the exponentially modified protein abundance index were the highest for PPP1CA, which showed that the probability of circGPRC5A interacting with PPP1CA was the greatest. The function PPP1CA protein and its role in cancer progression, especially for CRC, is unknown.

Based on the above results, we decided to explore the relationship between circGPRC5A and PPP1CA. To further confirm the binding relationship between circGPRC5A and PPP1CA, a RIP assay was conducted to verify that circGPRC5A was specifically precipitated with PPP1CA in HT29 and HCT116 cells, in contrast to the control IgG group (Fig. 5F, G). Next, we used immunofluorescence-FISH assays to visualize and identify the colocalization of circGPRC5A and PPP1CA in both the cytoplasm and nucleus of HT29 and HCT116 cells (Fig. 5H). In addition, we explored how circGPRC5A affects PPP1CA by knocking down or overexpressing circGPRC5A in HT29 and HCT116 cells. We surprisingly found that PPP1CA mRNA levels remained unchanged with circGPRC5A expression manipulation (Fig. 5I). However, PPP1CA protein levels changed (Fig. 5J–L, Figure S6A). These results indicate that circGPRC5A can stabilize PPP1CA at the protein level.

In order to further explore the relationship between circGPRC5A and PPP1CA and verify that circGPRC5A can stabilize PPP1CA at the protein level, we conducted PPP1CA protein level detection in 40 pairs of cancer and adjacent samples, which revealed that PPP1CA expression was higher in cancer tissues than in adjacent tissues (Figure S2A, B). Using correlation analysis, we also found a positive correlation between PPP1CA and circGPRC5A, indicating that circGPRC5A affected the protein level of PPP1CA (Figure S2C).

### CircGPRC5A inhibits PPP1CA and UBA1 binding and protects PPP1CA from proteasomal degradation

**Fig. 6:**
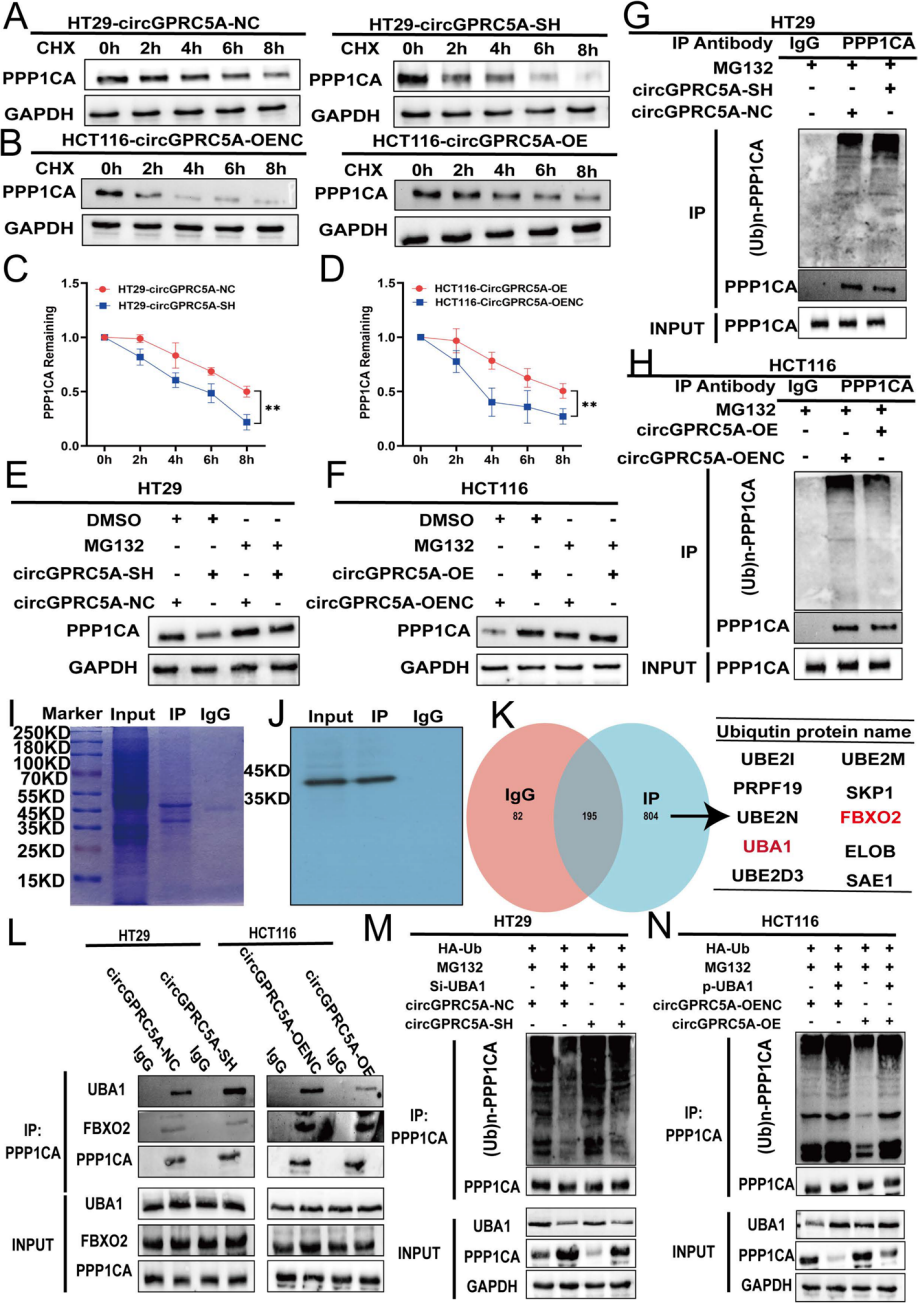
CircGPRC5A stabilizes PPP1CA protein by inhibiting the binding between UBA1 and PPP1CA. **A**–**D** After silencing or overexpressing circGPRC5A in HT29 and HCT116 cells, respectively, transfected CRC cells were treated with CHX (100 µg/mL) at the indicated times, and PPP1CA protein was evaluated using western blotting. **E**–**F** After silencing or overexpressing circGPRC5A in HT29 and HCT116 cells, respectively, transfected CRC cells were treated with MG132 (20 µM) for 6 h and then PPP1CA was evaluated using western blotting. **G**–**H** An IP assay was used to detect the polyubiquitination level of PPP1CA after silencing or overexpressing circGPRC5A. The immunocomplexes were examined using western blotting with anti-Ub and anti-PPP1CA antibodies.** I**-**J** PPP1CA was immunoprecipitated with an anti-PPP1CA antibody, and IgG was used as the negative control. The immunocomplexes were analyzed using western blotting, and then the SDS-PAGE gel was stained with Coomassie Brilliant Blue. **K** Mass spectrometry analysis was used to analyze the products of immunocomplexes immunoprecipitated with anti-PPP1CA antibody. Ubiquitination related proteins are shown. **L** CircRNA inhibited the binding between UBA1 and PPP1CA. The immunocomplexes with an anti-PPP1CA antibody were examined by western blotting. **M**–**N** CircGPRC5A inhibited PPP1CA ubiquitin levels by inhibiting the binding between UBA1 and PPP1CA. Immunocomplexes with an anti-PPP1CA antibody were assessed using the indicated antibodies by western blotting

Ubiquitination and de-ubiquitination play important roles in the homeostasis of protein modification and biological activities, and are dynamic because of imbalances of ubiquitination and de-ubiquitination [28, 29]. The potential function of circGPRC5A to de-ubiquitinate PPP1CA protein, thereby stabilizing it, is unknown. To assess whether circGPRC5A affects PPP1CA protein at the post-translational level or protein synthesis level, the degradation rate of PPP1CA was measured using the protein synthesis inhibitor cycloheximide (CHX, 100 µg/ml). Interestingly, compared to the controls, CHX treatment resulted in lower PPP1CA protein with circGPRC5A silencing, and higher protein with circGPRC5A overexpression (Fig. 6A–D). This further demonstrates that circGPRC5A can affect PPP1CA protein at the post-translational level. Therefore, we used MG132, which is a proteasome inhibitor that can effectively inhibit the ubiquitination process of proteins via proteasomal degradation. For HT29 silencing of the circGPRC5A group, the expression of PPP1CA was partially reversed after using MG132. In contrast, for HCT116 overexpressing the circGPRC5A group, the expression of PPP1CA was stable after using MG132. The results revealed that circGPRC5A affected the expression of PPP1CA via proteasomal degradation (Fig. 6E and F, Figure S6B and C). A co-IP assay also demonstrated that silencing circGPRC5A in CRC cells increased PPP1CA protein poly-ubiquitination levels (Fig. 6G, Figure S6D). In contrast, overexpression of circGPRC5A significantly reduced PPP1CA protein poly-ubiquitination (Fig. 6H, Figure S6E). In conclusion, these results reveal that circGPRC5A can stabilize PPP1CA protein by preventing ubiquitin-mediated proteasomal degradation.

We did not find that related de-ubiquitination proteins interacted with circGPRC5A and PPP1CA in the RNA pulldown products. Hence, we speculate that circGPRC5A may interfere with binding between PPP1CA and related ubiquitination proteins. To test this hypothesis, an IP assay for PPP1CA was conducted and the products from the IP were analyzed using mass spectrometry. The presence of ten related ubiquitination proteins were confirmed (Fig. 6I–K). Among these ten related ubiquitination proteins, UBA1 and FBXO2 could directly bind to PPP1CA, while the remaining proteins typically affected the levels of protein ubiquitination by forming ubiquitination complexes [30, 31]. We hypothesized that circGPRC5A interfered with the binding of PPP1CA to UBA1 or FBXO2, thereby affecting PPP1CA protein levels. The ability of UBA1 and PPP1CA to bind changed after circGPRC5A was silenced or overexpressed, but not for FBXO2, according to an IP for PPP1CA (Fig. 6L, Figure S6F and G). Furthermore, the partial reversal of circGPRC5A causing the change of PPP1CA poly-ubiquitination level was observed through silencing or overexpressing UBA1 (Fig. 6M and N, Figure S6J–M). Collectively, circGPRC5A can interfere with the binding between UBA1 and PPP1CA and further stabilize PPP1CA protein levels.

### PPP1CA and UBA1 are critical for circGPRC5A-mediated promotion of CRC

**Fig. 7:**
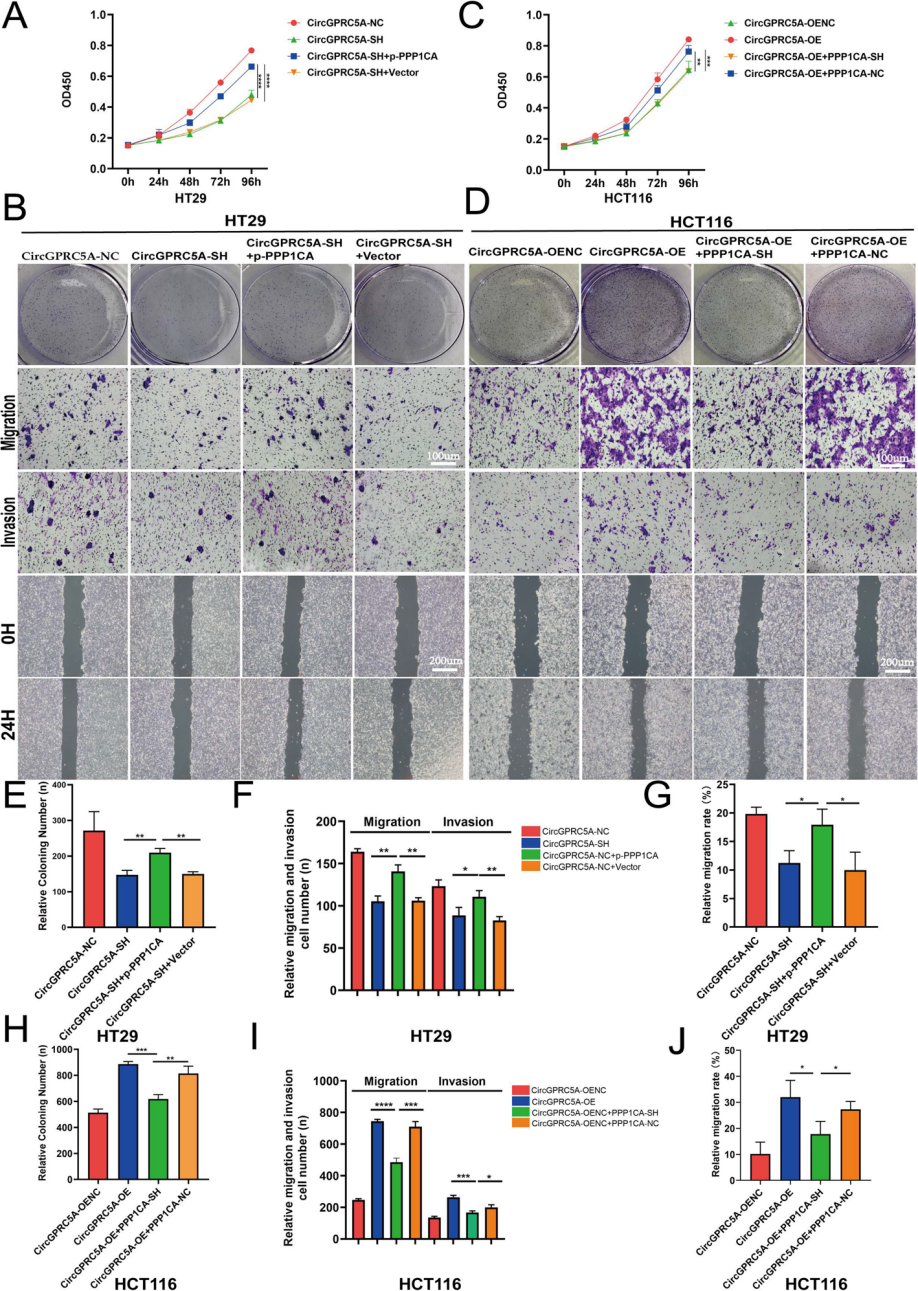
PPP1CA is critical for circGPRC5A-mediated promotion of CRC. **A**–**D** CCK-8, colony formation, Transwell, and wound healing assays were conducted to investigate the effects of PPP1CA on circGPRC5A-mediated proliferation and migration of HT29 and HCT116 cells. **E**–**J** Experimental results of the effects of PPP1CA on circGPRC5A-mediated proliferation and migration of HT29 and HCT116 cells were quantified and presented using a bar chart. Values are shown as the mean ± SD based on three independent experiments. *P < 0.05; **P < 0.01; ***P < 0.001

PPP1CA is frequently overexpressed in tumors, and some studies have shown that correlations with clinicopathological traits are linked to the emergence of malignant tumors [17, 18]. CCK-8, colony formation, Transwell, and wound healing assays were conducted to investigated the effects of PPP1CA on circGPRC5A-mediated proliferation and migration of CRC cells. These experiments aimed to determine whether circGPRC5A stabilizes PPP1CA to influence the proliferation and migration of CRC cells. The partial reversal of circGPRC5 silencing-induced inhibitory effects on cell proliferation and migration was observed through the overexpression of PPP1CA in HT29 cells (Fig. 7A, B and E-G). In contrast, circGPRC5A overexpression-induced cell proliferation and migration in HCT116 cells could be partially mitigated with PPP1CA knockdown (Fig. 7C, D and H–J). These results support the notion that PPP1CA is critical for circGPRC5A-mediated CRC progression.

In previous studies, we reported that UBA1 was a key protein for circGPRC5A to stabilize PPP1CA. Therefore, we continued to explore the role of UBA1 on circGPRC5A-mediated promotion of CRC. By using CCK-8, colony formation, Transwell, and wound healing assays, partial reversal of circGPRC5 silencing-induced inhibitory effects on cell proliferation and migration were observed using UBA1 knockdown in HT29 cells (Figure S3A, B, E–G). In addition, circGPRC5A overexpression-induced cell proliferation and migration in HCT116 cells could be partially mitigated with overexpression of UBA1 (Figure S3C, D and H–J).

### Impact of circGPRC5A on YAP-Ser127 and YAP-Ser109 dephosphorylation through stabilization of PPP1CA protein

**Fig. 8:**
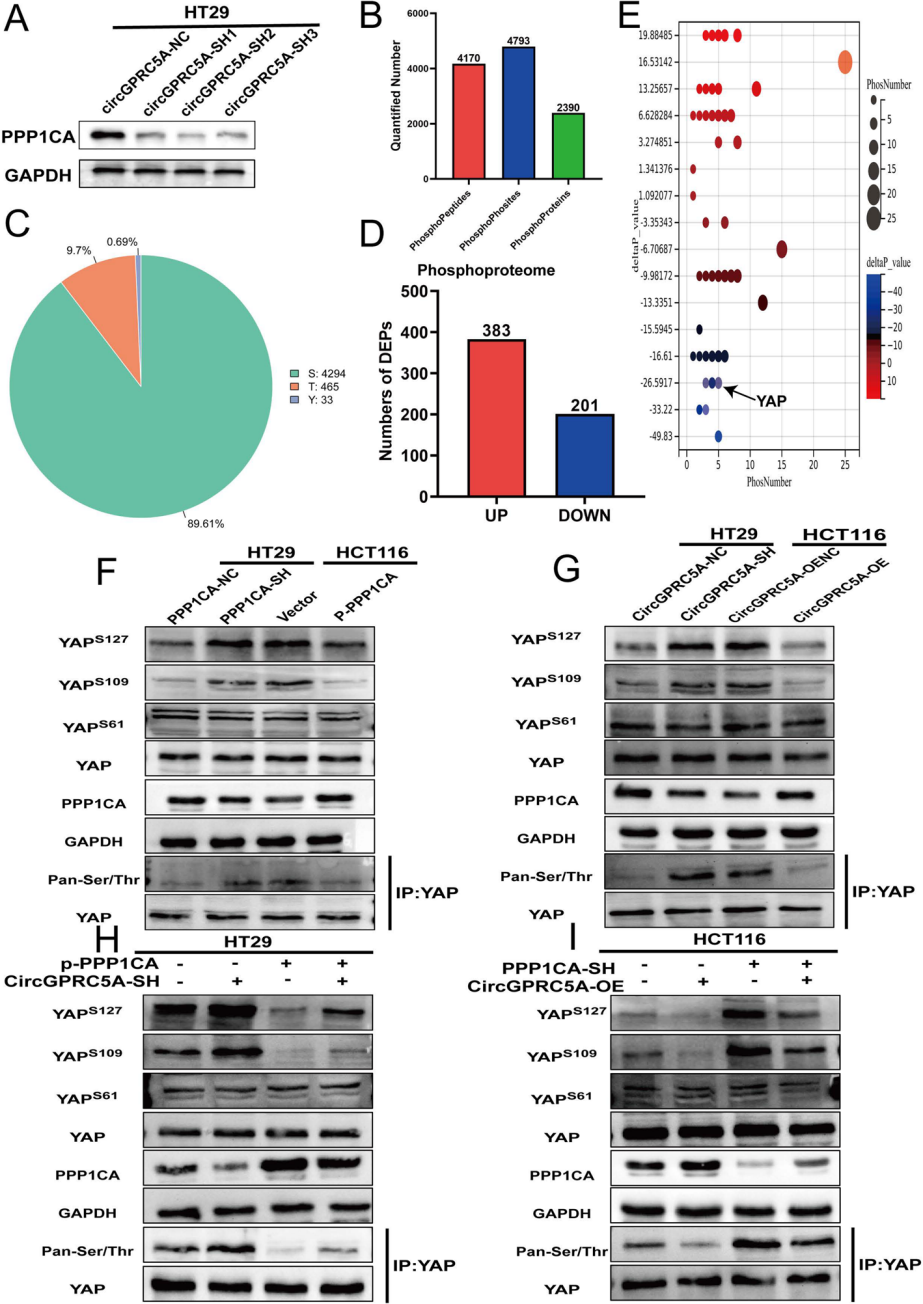
CircGPRC5A can cause dephosphorylation of YAP-ser127 and YAP-ser109 by stabilizing the PPP1CA protein. **A** Western blotting was used to examine PPP1CA protein after silencing circGPRC5A. **B** Phosphoproteomics revealed 4,170 phosphopeptides, 4,793 phosphorylation sites, and 2,390 phosphoproteins. **C** The pie chart illustrates the types of phosphorylated amino acids from quantitative phosphoproteomics analysis. **D** Upregulated and downregulated protein phosphorylation levels from quantitative phosphoproteomics analysis. **E** Bubble chart depicting the deltaP_value and phosphorylation site number. **F**,** G** Western blotting was used to examine YAP phosphorylation after silencing and overexpressing PPP1CA or circGPRC5A. **H**–**I** The effects of YAP dephosphorylation via PPP1CA on circGPRC5A-induced changes were detected by western blotting

We confirmed that circGPRC5A can stabilize PPP1CA, which is essential for circGPRC5A-mediated CRC progression. As a dephosphorylation protein, the imbalance of PPP1CA would inevitably lead to change of phosphorylation status, which could affect various biological behaviors. We hypothesized that circGPRC5A can lead to an imbalance in phosphorylation levels by stabilizing PPP1CA, thereby causing CRC progression (Fig. 8A). Phosphoproteomics was conducted to analyze the changes in phosphorylation states in circGPRC5A-silenced CRC cell lines. Phosphoproteomics revealed 4170 phosphopeptides, 4793 phosphorylation sites and 2390 phosphoproteins (Fig. 8B). Phosphoproteomics further demonstrated that PPP1CA mainly affected serine phosphorylation sites (89.61%), followed by threonine (9.7%) and lysine (0.69%) (Fig. 8C).

Phosphorylation enrichment results in the removal of most non-phosphorylated peptides, preventing accurate protein quantification. Therefore, based on the expression level and the number of phosphorylated peptides, a new variable called protein phosphorylation state value (deltaP_value) was defined. When deltaP_value ≥ 1, it indicates that the phosphorylated peptide in the protein is upregulated, and when deltaP_value ≤  − 1, it demonstrates that the phosphorylated peptide in the protein is downregulated. According to deltaP_value, we found upregulated phosphorylation levels of 383 proteins and downregulated phosphorylation levels of 201 proteins (Fig. 8D and E). Based on the deltaP_value and phosphorylation site number, we found that PPP1CA is most likely to lead to dephosphorylation of YAP and DBN1 proteins. YAP plays a pivotal role in cancer progression by changing phosphorylation [32]. DBN1 plays an important role in the formation of cell projections by changing expression [33, 34]. Although some studies have revealed that both YAP and DBN1 could participate in tumor progression, it is currently unclear whether changes in the phosphorylation status of DBN1 results in functional changes. Therefore, we inferred that circGPRC5A may lead to CRC progression by stabilizing PPP1CA and interfering with YAP phosphorylation levels.

The phosphoproteomics analysis revealed that YAP shows dephosphorylation at the following sites: Ser127, Ser138, Ser61, Ser109, Ser163/164 and Ser332. Dephosphorylation of YAP at Ser127 and Ser109 would cause YAP to enter the nucleus and bind to TEADs, thereby affecting cell proliferation [35, 36]. Phosphorylation of YAP-Ser61 may lead to transcriptional inhibition of YAP [37]. The role of other YAP phosphorylation sites is not clear. We next used co-IP assays to detect the total phosphorylation level of YAP. We found that silencing circGPRC5A or PPP1CA resulted in an increase in YAP phosphorylation levels. In addition, YAP phosphorylation of Ser127 and Ser109, but not Ser61, increased with silencing of circGPRC5A or PPP1CA (Fig. 8F, G, Figure S7A–J). We next investigated whether circGPRC5A can affect YAP phosphorylation levels by stabilizing PPP1CA. We found that the inhibiting effects on YAP dephosphorylation by silencing circGPRC5S was partially rescued after overexpression of PPP1CA in HT29 cells (Fig. 8H, Figure S7K–O). In HCT116 cells, circGPRC5A overexpression-induced YAP dephosphorylation could also be partially mitigated by knockdown of PPP1CA. (Fig. 8I, Figure S7P–T). We also extracted cytoplasmic and nuclear proteins separately and found that the change of YAP phosphorylation state led to changes in the distribution of YAP. PPP1CA-mediated dephosphorylation of YAP at Ser127 and Ser109 induced translocation of YAP in the nucleus (Figure S4A–D). These findings reveal that circGPRC5A may lead to CRC progression by interfering with YAP phosphorylation levels through stabilizing PPP1CA.

To further explore the important role of YAP on circGPRC5A/PPP1CA-mediated CRC progression, we demonstrated that the partial reversal of circGPRC5A/PPP1CA-mediated effects on cell proliferation and migration including CCK-8, colony formation, Transwell, and wound healing assays were observed using Verteporfin (YAP inhibitor, 10 ug/mL for 24 h) in HT29 and HCT116 cells (Figure S5A–J).

### CircGPRC5A promotes the proliferation and migration of CRC cells in vivo

**Fig. 9:**
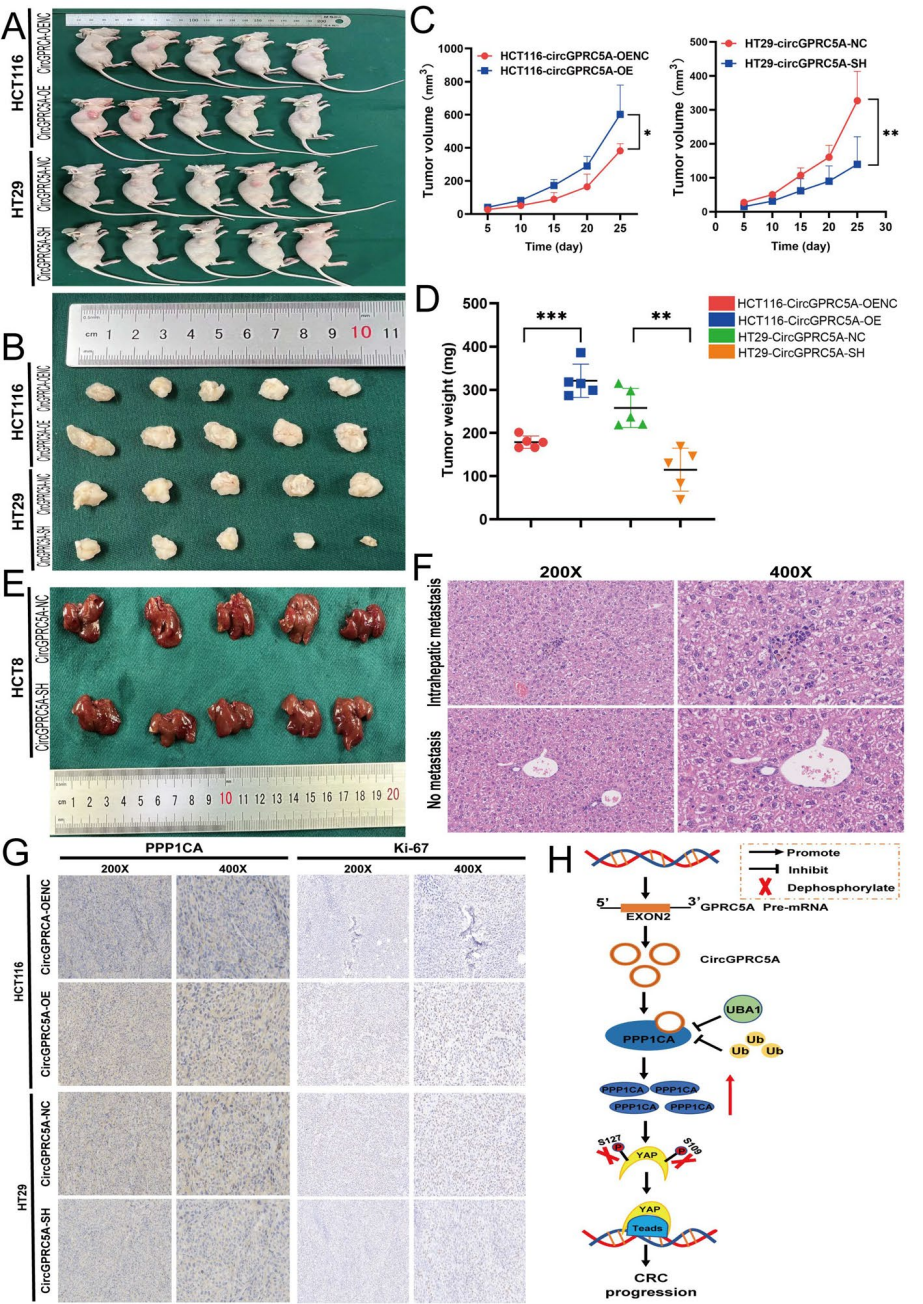
CircGPRC5A induces the growth of CRC cells in vivo. **A** and **B** Images of subcutaneous xenograft tumors. **C** After xenograft implantation, the xenograft tumor growth curve was assessed every 5 days. **D** Subcutaneous xenograft tumor weights. **E** The effect of knocking-down circGPRC5A on intrahepatic metastasis. **F** Images of hematoxylin-and-eosin-stained intrahepatic metastases. **G** Ki-67 and PPP1CA staining and IHC analysis of subcutaneous xenograft tumors. **H** Diagram illustrating the role of circGPRC5A in CRC

To verify the effect of circGPRC5A on CRC cell proliferation in vivo, circGPRC5A-knockdown and circGPRC5A-overexpression HT29 and HCT116 cells were xenografted subcutaneously into nude mice. The size of the xenograft tumors was measured on days 5, 10, 15, 20, and 25 after tumor cell injection. The circGPRC5A-overexpressing HCT116 tumors were the largest by volume and weight with the fastest tumor growth compared with the vector control group. On the other hand, knocking down circGPRC5A in HT29 cells resulted in smaller tumors by volume and weight, and the tumors were slower-growing (Fig. 9A–D).

Next, we explored the effect of circGPRC5A on CRC cell metastases. Female nude mice were injected with stably-transfected HCT8 cells with either circGPRC5A knockdown vector or the empty vector control. In contrast to the control group, we discovered that the circGPRC5A knockdown group had fewer metastatic nodules (Fig. 9E, F). IHC staining for Ki-67 and PPP1CA was performed on subcutaneous xenograft tumor tissues. Compared with the control group, Ki-67 and PPP1CA expression increased in tumors from the circGPRC5A-overexpressing group and decreased in tumors from the circGPRC5A-knockdown group (Fig. 9G, Figure S8A–C). Consistent with the in vitro experiments, xenograft tumor experiments revealed that circGPRC5A could promote CRC growth.

## Discussion

The incidence and fatality rates of CRC have been on the rise. Numerous circRNAs have been discovered thanks to advancements in genome-wide sequencing technology and circRNA-specific bioinformatics algorithms, and some of these circRNAs play a role in CRC. [38]. In our study, we demonstrated that circGPRC5A expression was significantly upregulated in human CRC. The tumor size, lymph node status, and TNM stage were all significantly correlated with high circGPRC5A expression. Silencing circGPRC5A inhibited the proliferation, migration and invasion of CRC cells while circGPRC5A overexpression promoted the proliferation, migration and invasion of CRC cells. Mechanistically, circGPRC5A interacts with PPP1CA and leads to the de-ubiquitination of PPP1CA by inhibiting the binding between UBA1 and PPP1CA. PPP1CA further dephosphorylates YAP at Ser127 and Ser109, which allows YAP to enter the nucleus and bind to TEADs, thereby leading to CRC progression. Ultimately, we first report that circGPRC5A promotes CRC progression by stabilizing PPP1CA and dephosphorylating YAP at Ser127 and Ser109.

CircGPRC5A (has_circ_0025506) has been reported to be expressed in tumors. One study revealed that circGPRC5A-peptide-GPRC5A can be utilized to target bladder cancer and bladder cancer stem cells [26]. Another study showed that circGPRC5A can bind miR-1283 and activate the YAP1/TEAD1 signaling pathway to promote the growth of hepatocellular carcinoma [27]. Similarly, by using circRNA expression profiling, we discovered that in contrast to nearby normal tissue, circGPRC5A was expressed at high levels in CRC. At the same time, higher expression of circGPRC5A is linked to tumor size, lymph node status, and TNM stage, further demonstrating that circGPRC5A may be a circRNA unique to CRC.

CircRNA-protein interactions play an essential role in multiple signaling pathways in cancer, such as protein expression, degradation, and biogenesis. Zheng et al. have reported that circLPAR1 exosomal direct interaction with eIF3h inhibited the METTL3-eIF3h interaction and reduced the translation of the oncogene BRD4 [39]. On the other hand, Xu et al. have reported that in clear cell renal cell carcinoma, circPOLR2A can bind to UBE3C and PEBP1 and degrade PEBP1, thereby inhibiting the ERK signaling pathway [40]. In our study, we revealed that circGPRC5A directly interacts with PPP1CA by using RNA pulldown and RIP assays. We further found that circGPRC5A can stabilize PPP1CA by protecting it from proteasomal degradation. PPP1CA is important for cell division, glycogen metabolism and protein synthesis by balancing protein phosphorylation levels. In addition, PPP1CA is mainly involved in dephosphorylation of serine/threonine, which is ubiquitous in many physiological functions. Sun et al. reported that USP11 could stabilize PPP1CA, which further increased ERK/MAPK activity and facilitated tumor growth and metastasis in CRC [41]. On the other hand, KIF18A could promote the malignant development of glioblastoma by binding to PPP1CA [42]. Other previous studies have also demonstrated that PPP1CA contributes to a worse prognosis in various tumors [17, 18]. In our study, we revealed that upregulated PPP1CA protein was caused by interacting with circGPRC5A, further leading to CRC progression.

Numerous physiological processes, including the cell cycle, differentiation, signal transduction, are tightly regulated by ubiquitination. Proteasomal degradation after ubiquitination mainly involves four processes. First, ubiquitin activating enzyme E1 activates ubiquitin molecules. Second, ubiquitin activating enzyme E1 transfers the activated ubiquitin molecules to ubiquitin binding enzyme E2. Third, ubiquitin ligase E3 transfers ubiquitin molecules binding to ubiquitin binding enzyme E2 to the target protein. Finally, ubiquitinated proteins are eventually broken-down by the proteasome [43, 44]. In our study, to assess whether circGPRC5A affected PPP1CA protein at the protein degradation level or protein synthesis level, we used MG132, which is a proteasome inhibitor that can effectively inhibit the ubiquitination process of proteins via proteasomal degradation. We found that low circGPRC5A resulted in a decrease in the PPP1CA via proteasomal degradation, so MG132could effectively reverse this process. In contrast, high circGPRC5A inhibited PPP1CA degradation when using the MG132, and may not be effective. This result further revealed that circGPRC5A could stabilize the level of PPP1CA protein by proteasomal degradation. CircRNA can also participate in the process of ubiquitination, and some studies have shown that circRNA can bind to target proteins, thereby affecting protein degradation. Li et al. have reported that circINSIG1 can encode a 121 amino-acid protein circINSIGI-121 to promote ubiquitination of INSIG1 by recruiting the CUL5-ASB6 complex [45]. On the other hand, circRERE can modulate osteoarthritis by regulating β-catenin ubiquitination and degradation [46]. In our study, a co-IP assay revealed that circGPRC5A can affect the binding between PPP1CA and UBA1 to further stabilize PPP1CA. UBA1 can mark cellular proteins for degradation through the ubiquitin–proteasome system, which can lead to the development of various diseases including small-cell lung cancer and VEXAS syndrome [47, 48]. Our results suggest that circGPRC5A may occupy the binding site of UBA1 on PPP1CA, resulting in UBA1 unable to mark PPP1CA, thereby increasing the PPP1CA protein level.

Phosphorylation is a kind of post-translational modification and phosphorylated proteins account for about one-third of all proteins [49]. Therefore, phosphorylation can affect numerous processes such as intracellular signal transduction, cell structure, cell proliferation, and apoptosis [50]. The Hippo signaling pathway can serve as a typical representative of phosphorylation modification and take part in various physiological activities. YAP plays a critical role in the hippo pathway and its imbalance in YAP phosphorylation can lead to the progression of various diseases. CircRNAs can also contribute to tumor progression by affecting the expression and phosphorylation of YAP. Zheng et al. have reported that circPPP1R12A can encode a novel protein to promote tumor progress via Hippo-YAP signaling [51]. On the other hand, circRNA_104075 can regulate HNF4A to further stimulate YAP-dependent tumorigenesis in hepatocellular carcinoma [52]. We found that circGPRC5A may lead to CRC progression by interfering with YAP phosphorylation of Ser127 and Ser109, which stabilizes PPP1CA. These data reiterate that circRNA can participated in the phosphorylation process and play a significant role in tumor progression.

In conclusion, the research shows that circGPRC5A functions as a tumor-promoting gene to encourage CRC cell growth and metastasis both in vivo and in vitro. CircGPRC5A is highly correlated with clinicopathological characteristics of CRC patients, such as tumor size, lymph node status, and TNM stage. Mechanistically, circGPRC5A acts as a binding molecule to enhance the levels of PPP1CA protein by preventing the interaction between PPP1CA and UBA1. As a result, it leads to the dephosphorylation of YAP at Ser127 and Ser109. The dephosphorylation of YAP at Ser127 and Ser109 results in YAP entering the cell nucleus and binding to TEADs, leading to CRC progression. Thus, our study revealed that circGPRC5A plays an essential role in CRC progression and can act as a new and potential target for patients with CRC.

### Supplementary Information

**Additional file 1: Figure S1. **CircGPRC5A expression in paired normal tissues and CRC tissues by using a FISH probe. A, B FISH was used to investigate the expressions of circGPRC5A in paired normal tissues and CRC tissues. C Statistical analysis using a bar chart to evaluate relative expression of circGPRC5A.v. **Figure S2. **The relationship between PPP1CA and circGPRC5A. A, B PPP1CA protein level detection in 40 pairs of cancer and adjacent samples using western blotting. C A positive correlation between PPP1CA and circGPRC5A using correlation analysis. **Figure S3.** UBA1 is critical for circGPRC5A-mediated promotion of CRC. A–D CCK-8, colony formation, Transwell, and wound healing assays were conducted to investigate the effects of PPP1CA on circGPRC5A-mediated proliferations and migrations of HT29 and HCT116 cells. E –J The experimental results involving the effects of UBA1 on circGPRC5A-mediated proliferation and migration of HT29 and HCT116 cells were quantified and presented using a bar chart. Values are shown as the mean ± SD based on three independent experiments. *P < 0.05; **P < 0.01; ***P < 0.001. **Figure S4.** CircGPRC5A can cause dephosphorylation of YAP and further lead to changes in the distribution of YAP by stabilizing the PPP1CA protein. A, B Western blotting was used to examine nuclear YAP and total YAP after silencing and overexpressing PPP1CA or circGPRC5A. C**–**D The effects of YAP distribution via PPP1CA on circGPRC5A-induced changes were detected by western blotting. Values are shown as the mean ± SD based on three independent experiments. *P < 0.05;**P < 0.01; ***P < 0.001. **Figure S5. **YAP is critical for circGPRC5A/PPP1CA-mediated promotion of CRC. A, B CCK-8 assays were conducted to determine the effects of YAP on circGPRC5A/PPP1CA-mediated cell proliferation. C**–**J The effects of YAP on circGPRC5A/PPP1CA-induced cell proliferation and migration were measured with a colony formation assay, and migration and invasion were investigated using Transwell and wound healing assays. Values are shown as the mean ± SD based on three independent experiments. *P < 0.05;**P < 0.01; ***P < 0.001. **Figure S6.** Statistic analysis chart of western blotting for various proteins. A. PPP1CA protein levels were assessed after silencing or overexpressing circGPRC5A. B, C After silencing circGPRC5A in HT29 and overexpressing circGPRC5A in HCT116, PPP1CA was evaluated with or without MG132. D, E Ploy(Ub) for PPP1CA was assessed after silencing or overexpressing circGPRC5A in HT29 or HCT116. F, G UBA1 and FBXO2 protein levels were assessed after silencing or overexpressing circGPRC5A using the IP assay. H-K: PPP1CA and UBA1 were evaluated when silencing circGPRC5A and UBA1 in HT29 or overexpressing circGPRC5A and UBA1 in HCT116 cells. L**-**M: Ploy(Ub) for PPP1CA were evaluated by IP assays for PPP1CA when silencing circGPRC5A and UBA1 in HT29 or overexpressing circGPRC5A and UBA1 in HCT116 cells. Values from three independent experiments are presented. *P < 0.05; **P < 0.01;***P < 0.001. **Figure S7.** Statistic analysis chart of western blotting for various proteins. A-E: The protein levels of YAP^ser127^, YAP^ser109^, YAP^ser61^, PPP1CA, and YAP^pan ser/thr^ were assessed after overexpressing and silencing PPP1CA. F-G: The protein levels of YAP^ser127^, YAP^ser109^, YAP^ser61^, PPP1CA, and YAP^pan ser/thr^ were assessed after overexpressing and silencing circGPRC5A. K–O The protein levels of YAP^ser127^, YAP^ser109^, YAP^ser61^, PPP1CA, and YAP^pan ser/thr^ were assessed after silencing circGPRC5A and overexpressing PPP1CA in HT29 cells. P –T The protein levels of YAP^ser127^, YAP^ser109^, YAP^ser61^, PPP1CA, and YAP^pan ser/thr^ were assessed after overexpressing circGPRC5A and silencing PPP1CA in HCT116 cells. Values from three independent experiments are presented. *P < 0.05;**P < 0.01; ***P < 0.001. **Figure S8.** Statistic analysis chart of Ki-67 and PPP1CA staining and IHC analysis. A Quantitative analysis of liver metastases in mice. B Quantitative analysis of IHC for PPP1CA of subcutaneous xenograft tumors. C Quantitative analysis of IHE for Ki-67 of subcutaneous xenograft tumors.

**Additional file 2. **

**Additional file 3. **

**Additional file 4: Table S1. **shRNA, siRNA target sequences. **Table S2. **The primer sequences used for real-time quantitative PCR. **Table S3. **The sequence of probes was used in this study. **Table S4. **Association between the status of circGPRC5A expression and clinicopathological characteristics of human colorectal cancer.

## Data Availability

All data generated or analyzed during this study are included in this published article/supplementary material. Further inquiries can be directed to the corresponding author.

## Abbreviations

CRC: Colorectal cancer
CircRNAs: Circular RNAs
MicroRNA: MiRNA
CCK8: Cell Counting Kit-8
Edu: 5-Ethynyl-2′-deoxyuridine
FISH: Fluorescence in situ hybridization
RIP: RNA-binding protein immunoprecipitation
IP: Immunoprecipitation
IHC: Immunohistochemistry
qRT-PCR: Real-time quantitative polymerase chain reaction
SD: Standard deviation
