# Supplementary material for: CircGPRC5A enhances colorectal cancer progress by stabilizing PPP1CA and inducing YAP dephosphorylation
Source: J Exp Clin Cancer Res. 2023 Dec 6;42:334. doi: 10.1186/s13046-023-02915-7 (PMC10698990; doi:10.1186/s13046-023-02915-7)
Supplement: Supplementary file 1 — Additional file 1: Figure S1. CircGPRC5A expression in paired normal tissues and CRC tissues by using a FISH probe. A, B FISH was used to investigate the expressions of circGPRC5A in paired normal tissues and CRC tissues. C Statistical analysis using a bar chart to evaluate relative expression of circGPRC5A.v. Figure S2. The relationship between PPP1CA and circGPRC5A. A, B PPP1CA protein level detection in 40 pairs of cancer and adjacent samples using western blotting. C A positive correlation between PPP1CA and circGPRC5A using correlation analysis. Figure S3. UBA1 is critical for circGPRC5A-mediated promotion of CRC. A–D CCK-8, colony formation, Transwell, and wound healing assays were conducted to investigate the effects of PPP1CA on circGPRC5A-mediated proliferations and migrations of HT29 and HCT116 cells. E –J The experimental results involving the effects of UBA1 on circGPRC5A-mediated proliferation and migration of HT29 and HCT116 cells were quantified and presented using a bar chart. Values are shown as the mean ± SD based on three independent experiments. *P < 0.05; **P < 0.01; ***P < 0.001. Figure S4. CircGPRC5A can cause dephosphorylation of YAP and further lead to changes in the distribution of YAP by stabilizing the PPP1CA protein. A, B Western blotting was used to examine nuclear YAP and total YAP after silencing and overexpressing PPP1CA or circGPRC5A. C–D The effects of YAP distribution via PPP1CA on circGPRC5A-induced changes were detected by western blotting. Values are shown as the mean ± SD based on three independent experiments. *P < 0.05;**P < 0.01; ***P < 0.001. Figure S5. YAP is critical for circGPRC5A/PPP1CA-mediated promotion of CRC. A, B CCK-8 assays were conducted to determine the effects of YAP on circGPRC5A/PPP1CA-mediated cell proliferation. C–J The effects of YAP on circGPRC5A/PPP1CA-induced cell proliferation and migration were measured with a colony formation assay, and migration and invasion were investigated using Transwell and w [file 13046_2023_2915_MOESM1_ESM.docx]

**
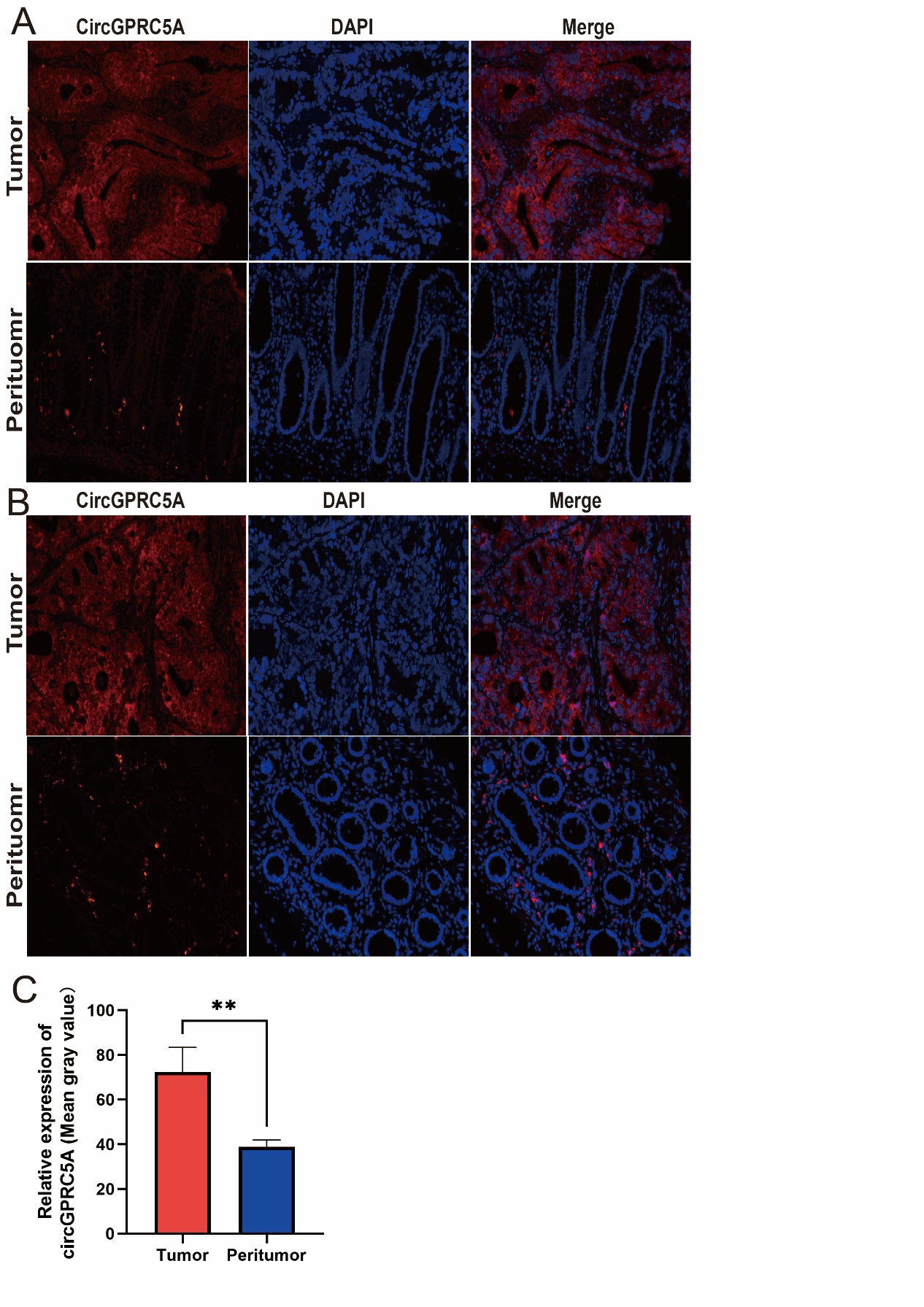
 Figure S1:** CircGPRC5A expression in paired normal tissues and CRC tissues by using a FISH probe. **A-B:** FISH was used to investigate the expressions of circGPRC5A in paired normal tissues and CRC tissues. **C:** Statistical analysis using a bar chart to evaluate relative expression of circGPRC5A.


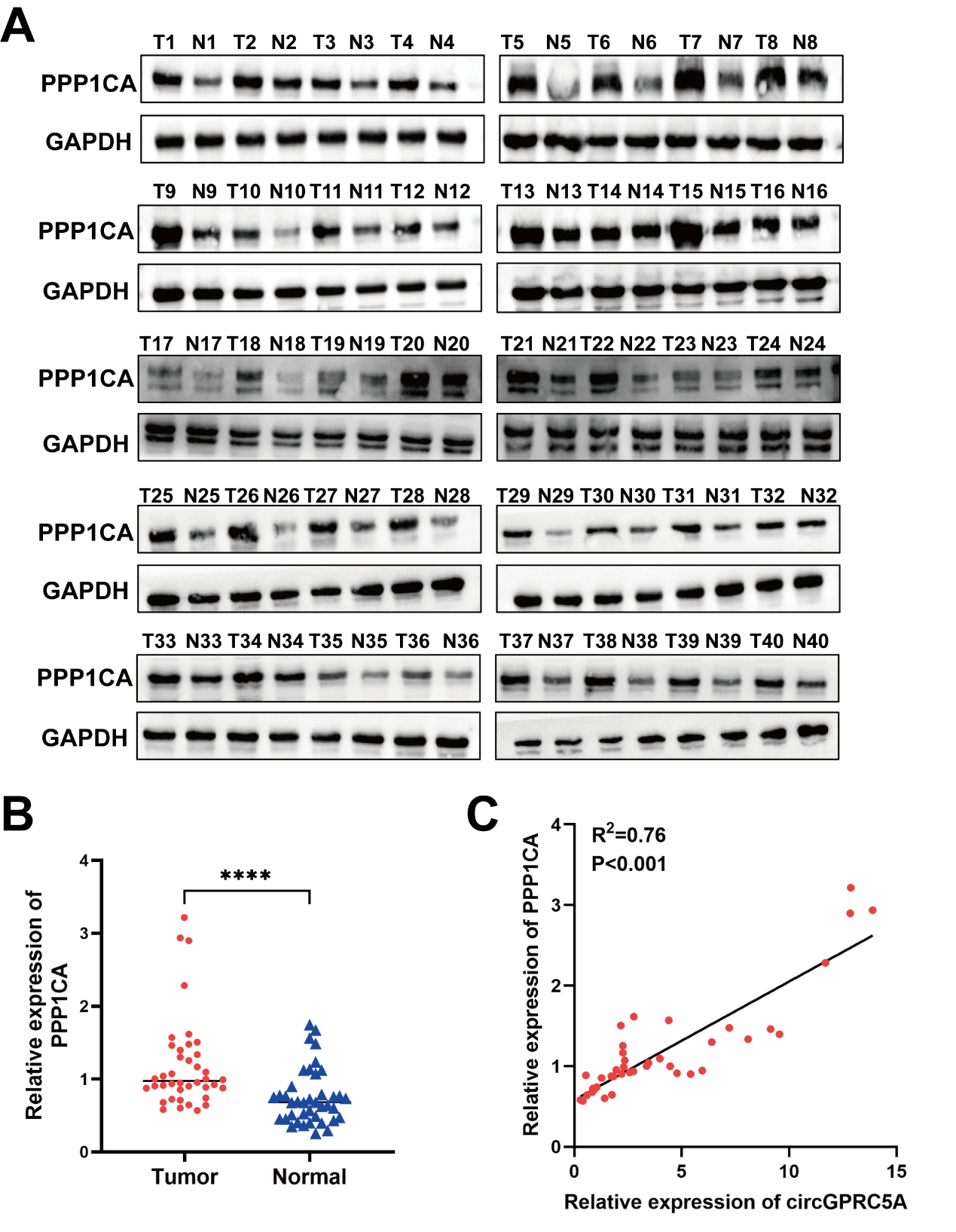


**Figure S2:** The relationship between PPP1CA and circGPRC5A. **A-B:** PPP1CA protein level detection in 40 pairs of cancer and adjacent samples using western blotting. **C:** A positive correlation between PPP1CA and circGPRC5A using correlation analysis.


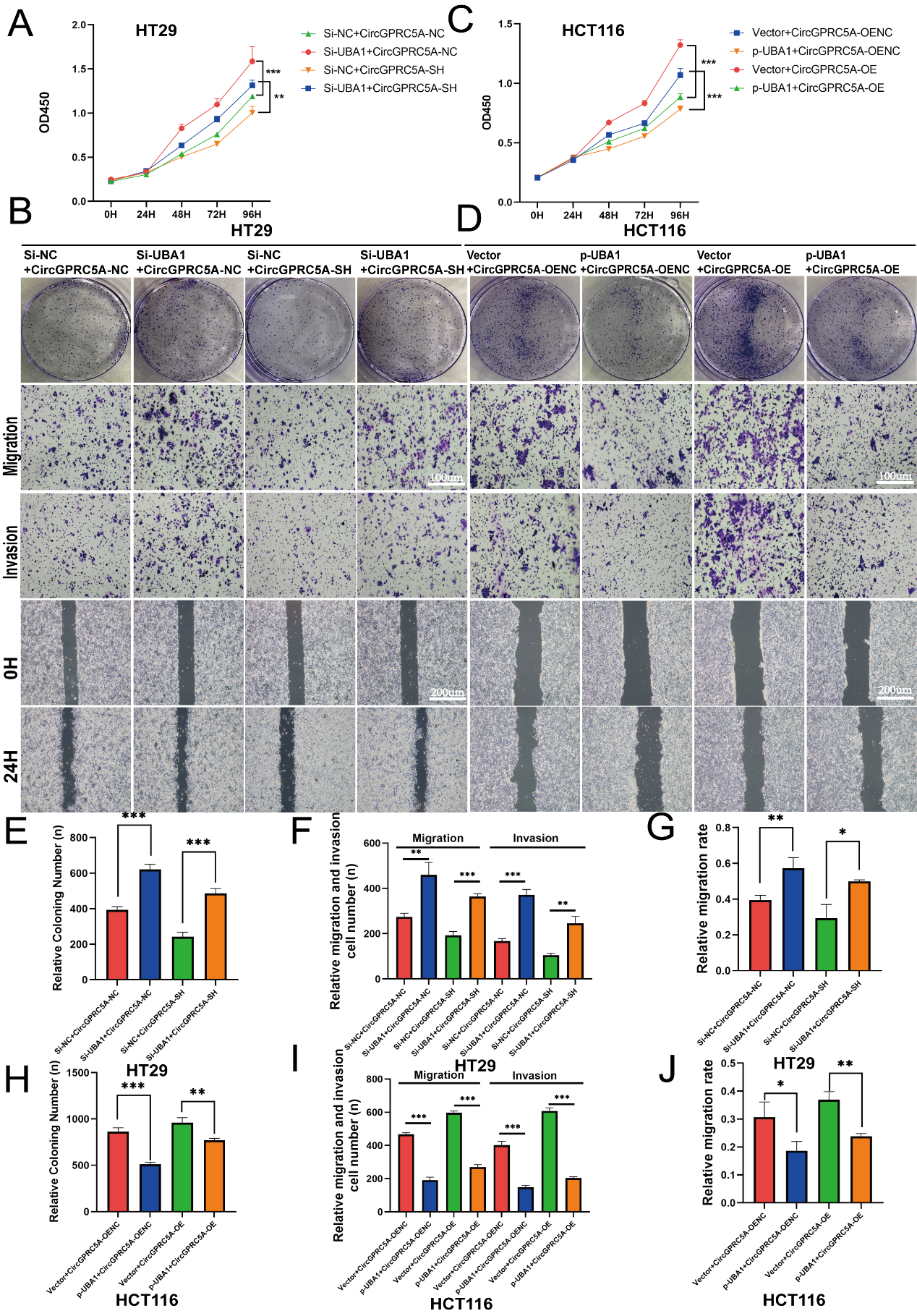


**Figure S3:** UBA1 is critical for circGPRC5A-mediated promotion of CRC. **A–D:** CCK-8, colony formation, Transwell, and wound healing assays were conducted to investigate the effects of PPP1CA on circGPRC5A-mediated proliferations and migrations of HT29 and HCT116 cells. **E-J:** The experimental results involving the effects of UBA1 on circGPRC5A-mediated proliferation and migration of HT29 and HCT116 cells were quantified and presented using a bar chart. Values are shown as the mean ± SD based on three independent experiments. *P < 0.05; **P < 0.01; ***P < 0.001.

**
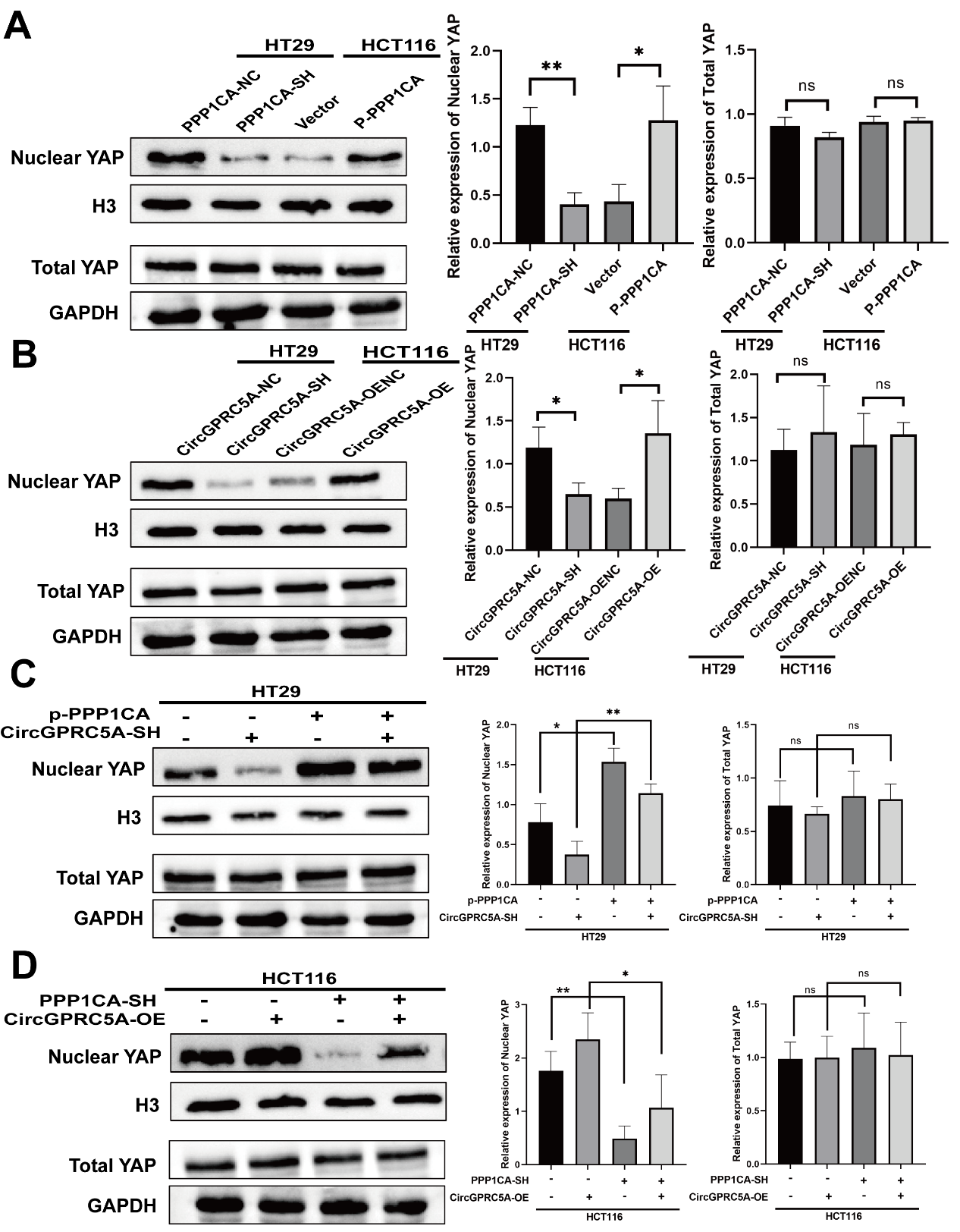
**

**Figure S4:** CircGPRC5A can cause dephosphorylation of YAP and further lead to changes in the distribution of YAP by stabilizing the PPP1CA protein. **A-B:** Western blotting was used to examine nuclear YAP and total YAP after silencing and overexpressing PPP1CA or circGPRC5A. **C-D:** The effects of YAP distribution via PPP1CA on circGPRC5A-induced changes were detected by western blotting. Values are shown as the mean ± SD based on three independent experiments. *P < 0.05; **P < 0.01; ***P < 0.001.


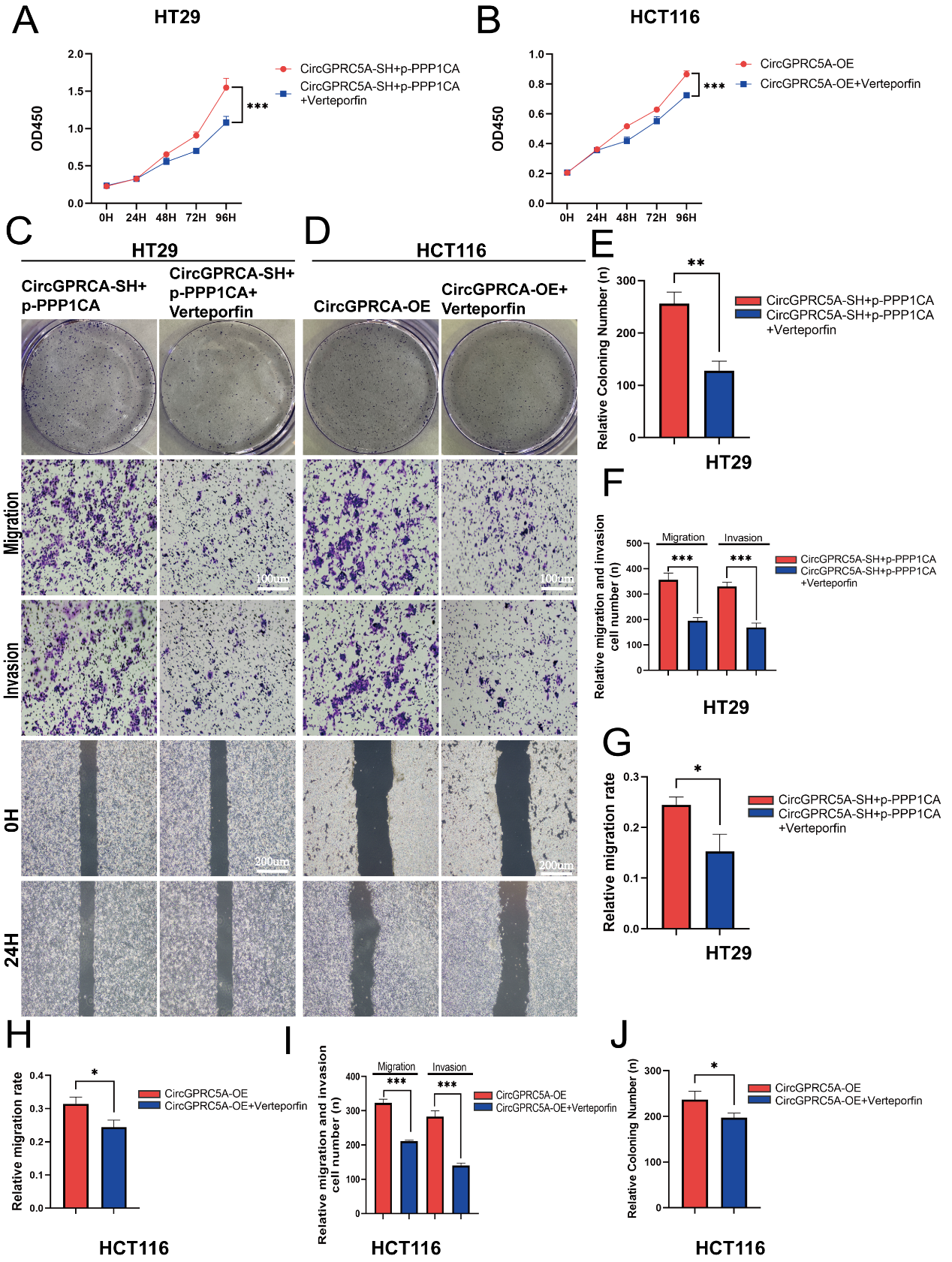


**Figure S5:** YAP is critical for circGPRC5A/PPP1CA-mediated promotion of CRC. **A-B:** CCK-8 assays were conducted to determine the effects of YAP on circGPRC5A/PPP1CA-mediated cell proliferation. **C-J:** The effects of YAP on circGPRC5A/PPP1CA-induced cell proliferation and migration were measured with a colony formation assay, and migration and invasion were investigated using Transwell and wound healing assays. Values are shown as the mean ± SD based on three independent experiments. *P < 0.05; **P < 0.01; ***P < 0.001.


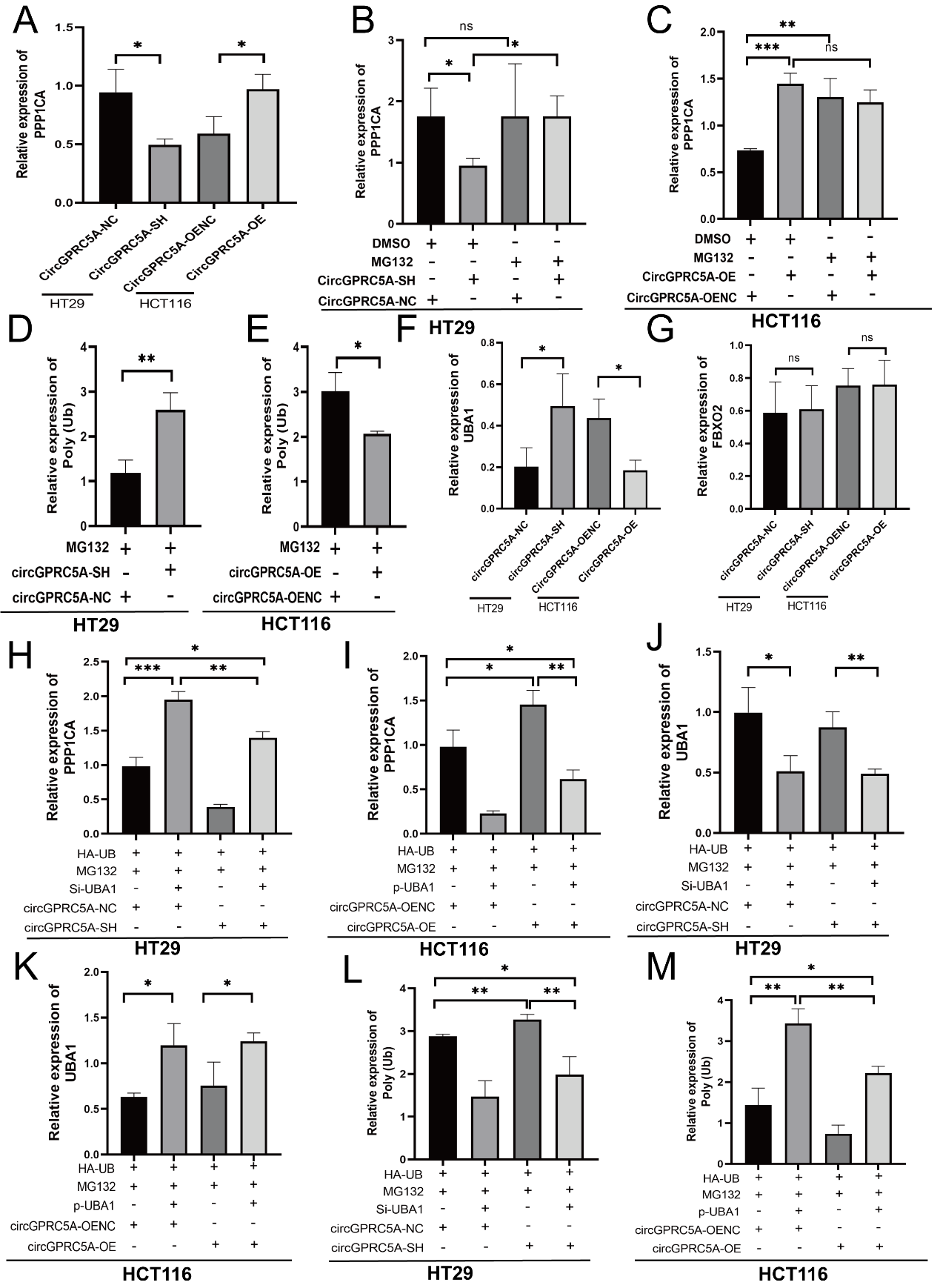


**Figure S6: Statistic analysis chart of western blotting for various proteins. A.** PPP1CA protein levels were assessed after silencing or overexpressing circGPRC5A. **B-C.** After silencing circGPRC5A in HT29 and overexpressing circGPRC5A in HCT116, PPP1CA was evaluated with or without MG132. **D-E:** Ploy(Ub) for PPP1CA was assessed after silencing or overexpressing circGPRC5A in HT29 or HCT116. **F-G:** UBA1 and FBXO2 protein levels were assessed after silencing or overexpressing circGPRC5A using the IP assay. **H-K:** PPP1CA and UBA1 were evaluated when silencing circGPRC5A and UBA1 in HT29 or overexpressing circGPRC5A and UBA1 in HCT116 cells. **L-M:** Ploy(Ub) for PPP1CA were evaluated by IP assays for PPP1CA when silencing circGPRC5A and UBA1 in HT29 or overexpressing circGPRC5A and UBA1 in HCT116 cells. Values from three independent experiments are presented. *P < 0.05; **P < 0.01; ***P < 0.001.


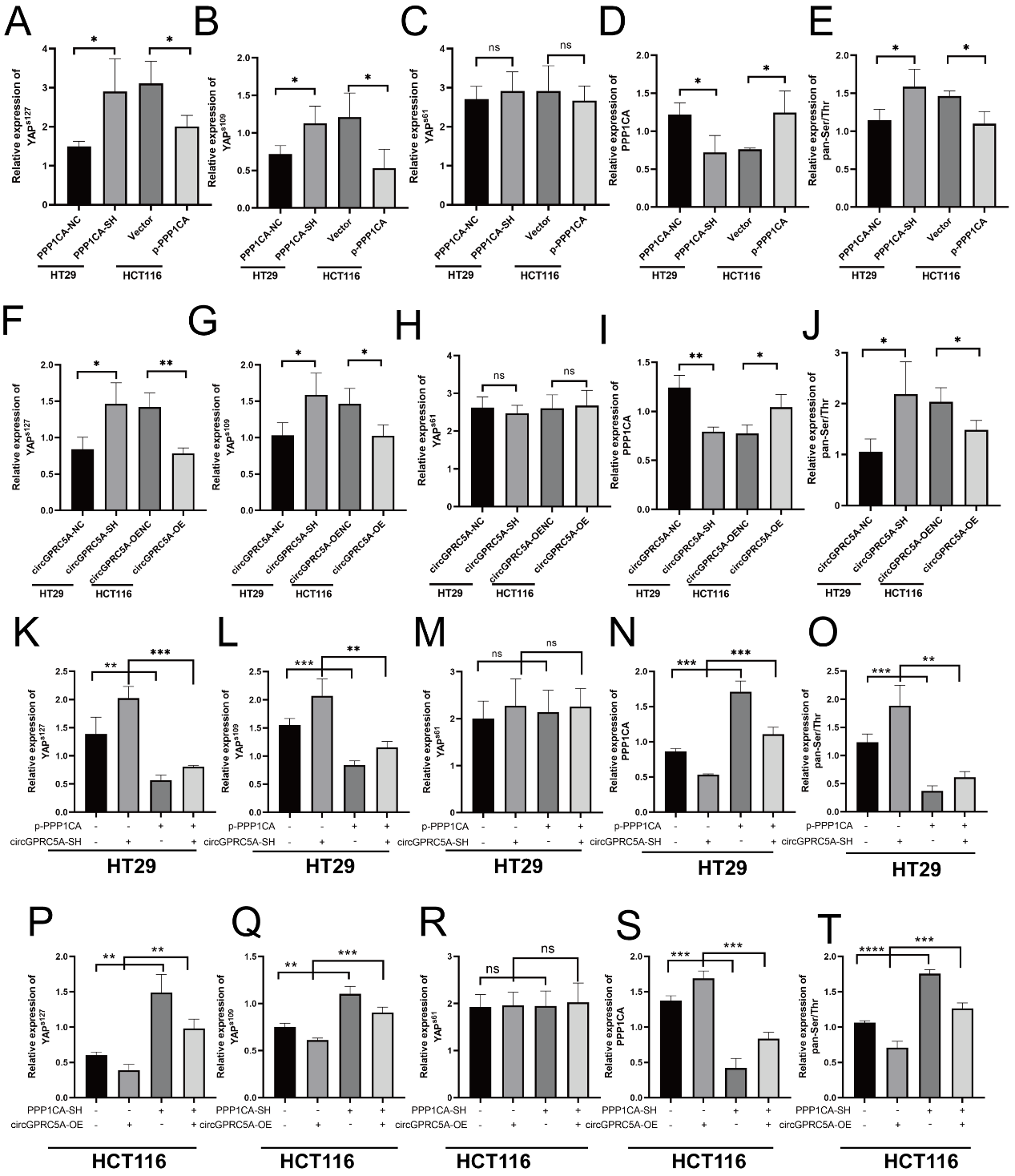


**Figure S7: Statistic analysis chart of western blotting for various proteins. A-E:** The protein levels of YAP^ser127^, YAP^ser109^, YAP^ser61^, PPP1CA, and YAP^pan ser/thr^ were assessed after overexpressing and silencing PPP1CA. **F-G:** The protein levels of YAP^ser127^, YAP^ser109^, YAP^ser61^, PPP1CA, and YAP^pan ser/thr^ were assessed after overexpressing and silencing circGPRC5A. **K-O:** The protein levels of YAP^ser127^, YAP^ser109^, YAP^ser61^, PPP1CA, and YAP^pan ser/thr^ were assessed after silencing circGPRC5A and overexpressing PPP1CA in HT29 cells. **P-T:** The protein levels of YAP^ser127^, YAP^ser109^, YAP^ser61^, PPP1CA, and YAP^pan ser/thr^ were assessed after overexpressing circGPRC5A and silencing PPP1CA in HCT116 cells. Values from three independent experiments are presented. *P < 0.05; **P < 0.01; ***P < 0.001.

**
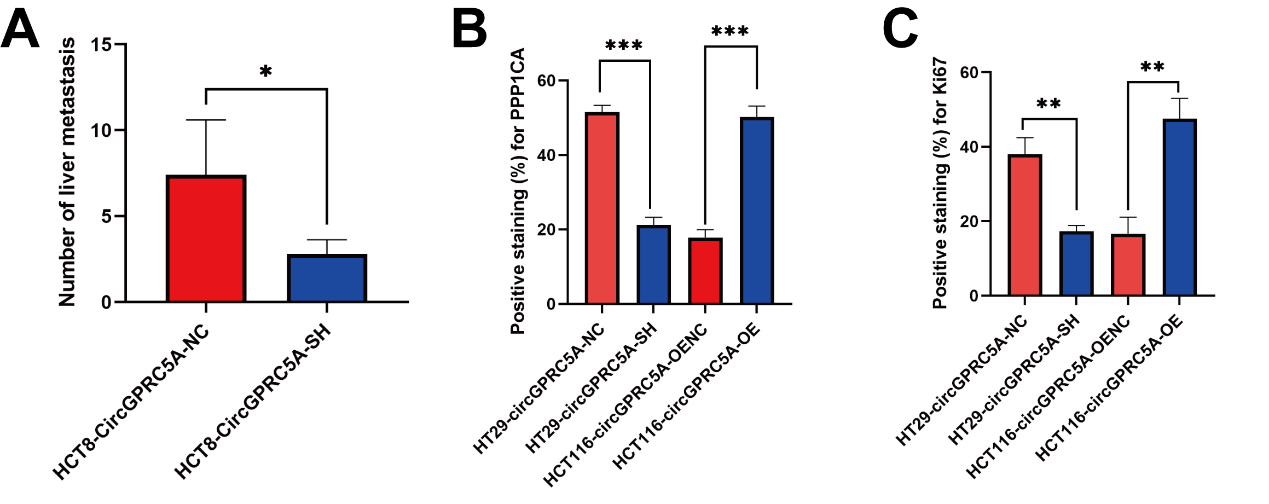
**

**Figure S8: Statistic analysis chart of Ki-67 and PPP1CA staining and IHC analysis. A:** Quantitative analysis of liver metastases in mice. **B:** Quantitative analysis of IHC for PPP1CA of subcutaneous xenograft tumors. **C:** Quantitative analysis of IHE for Ki-67 of subcutaneous xenograft tumors.
