# Supplementary material for: CircGPRC5A enhances colorectal cancer progress by stabilizing PPP1CA and inducing YAP dephosphorylation
Source: J Exp Clin Cancer Res. 2023 Dec 6;42:334. doi: 10.1186/s13046-023-02915-7 (PMC10698990; doi:10.1186/s13046-023-02915-7)
Supplement: Supplementary file 4 — Additional file 4: Table S1. shRNA, siRNA target sequences. Table S2. The primer sequences used for real-time quantitative PCR. Table S3. The sequence of probes was used in this study. Table S4. Association between the status of circGPRC5A expression and clinicopathological characteristics of human colorectal cancer. [file 13046_2023_2915_MOESM4_ESM.docx]

**Table S1. shRNA, siRNA target sequences**

| Gene name | Source | Target sequences |
| --- | --- | --- |
| Si-circGPRC5A#1 | RIBOBIO | 5’-GGAAATCACTCAAGGTCCA-3’ |
| Si-circGPRC5A#2 | RIBOBIO | 5’-ATCACTCAAGGTCCAGAAT-3’ |
| Si-circGPRC5A#3 | RIBOBIO | 5’-CACTCAAGGTCCAGAATGG-3’ |
| Sh-circGPRC5A | HANBIO | Top strand:  GATCCGATCACTCAAGGTCCAGAATCTCGAGATTCTGGACCTTGAGTGATTTTTTTG |
|  |  | Bottom strand:  AATTCAAAAAAATCACTCAAGGTCCAGAATCTCGAGATTCTGGACCTTGAGTGATCG |
| Sh-PPP1CA#1 | HANBIO | Top strand:  GATCCGACTACGACCTTCTGCGACTATCTCGAGATAGTCGCAGAAGGTCGTAGTTTTTTTG |
|  |  | Bottom strand:  AATTCAAAAAAACTACGACCTTCTGCGACTATCTCGAGATAGTCGCAGAAGGTCGTAGTCG |
| Sh-PPP1CA#2 | HANBIO | Top strand:  GATCCGTGAGTGCAAGAGACGCTACAACTCGAGTTGTAGCGTCTCTTGCACTCATTTTTTG |
|  |  | Bottom strand:  AATTCAAAAAATGAGTGCAAGAGACGCTACAACTCGAGTTGTAGCGTCTCTTGCACTCACG |
| Sh-PPP1CA#3 | HANBIO | Top strand:  GATCCGCTGCTGGCCTATAAGATCAACTCGAGTTGATCTTATAGGCCAGCAGCTTTTTTG |
|  |  | Bottom strand:  AATTCAAAAAAGCTGCTGGCCTATAAGATCAACTCGAGTTGATCTTATAGGCCAGCAGCG |
| Si-UBA1#1 | TSINGKE | Sence：  5’- CCUCCAACUUCUCCGACUA(dT)-3’ |
|  |  | Antisence:  5’- UAGUCGGAGAAGUUGGAGG(dT)-3’ |
| Si-UBA1#2 | TSINGKE | Sence：  5’-GACACCAUUGAGAAGUCAA(dT)-3’ |
|  |  | Antisence:  5’-UUGACUUCUCAAUGGUGUC(dT)-3’ |
| Si-UBA1#3 | TSINGKE | Sence：  5’- GGAUCGCUUUGAGGUACAA(dT)-3’ |
|  |  | Antisence:  5’- UUGUACCUCAAAGCGAUCC(dT)-3’ |

**Table S2. The primer sequences used for real-time quantitative PCR**

| Gene name |  | The primer sequences | Amplification  length (bp) |
| --- | --- | --- | --- |
| CircGPRC5A | Forward | TCCTGTTGAGGATGCTTTCT | 108 |
|  | Reverse | TGTAGCCATTCTGGACCTTG |  |
| GPRC5A | Forward | CTCACTCTCCCGATCCTCGT | 129 |
|  | Reverse | CAGTCCGATGATGAAGGCGAA |  |
| PPP1CA | Forward | ATCTGCGGTGACATACACG | 113 |
|  | Reverse | TTGCCCCTGTCCACATAGT |  |
| UBA1 | Forward | CACAACTTCCCTCCTGACC | 125 |
|  | Reverse | GCAGCCATCACATAGTCCA |  |
| GAPDH | Forward | CTTTGGTATCGTGGAAGGACTC | 132 |
|  | Reverse | GTAGAGGCAGGGATGATGTTCT |  |

**Table S3. The sequence of probes was used in this study**

| Gene name | The probe sequence |
| --- | --- |
| CircGPRC5A | 5’-GUUGUAGCCAUUCUGGACCUUGAGUGAUUUC-3’ |

**Table S4. Association between the status of circGPRC5A expression and clinicopathological characteristics of human colorectal cancer**

| **Characteristics** | **No. of patients** | **circGPRC5A** | | **P value** |
| --- | --- | --- | --- | --- |
|  |  | **High** | **Low** |  |
| **All cases** | 80 | 48 | 32 |  |
| **Gender** |  |  |  |  |
| Male | 47 | 28 | 19 | 0.93 |
| Female | 33 | 20 | 13 |  |
| **Age** |  |  |  |  |
| ≥60 | 53 | 32 | 21 | 0.92 |
| <60 | 27 | 16 | 11 |  |
| **Tumor size(cm)** |  |  |  |  |
| ≤5 | 48 | 23 | 25 | 0.01 |
| >5 | 32 | 25 | 7 |  |
| **Differentiation grade** |  |  |  |  |
| Well/Moderate differentiation | 46 | 26 | 20 | 0.46 |
| Poor differentiation | 34 | 22 | 12 |  |
| **Tumor location** |  |  |  |  |
| Right colon | 17 | 8 | 9 | 0.16 |
| Left colon | 21 | 16 | 5 |  |
| Rectum | 42 | 24 | 18 |  |
| **Lymph node status** |  |  |  |  |
| Positive | 37 | 27 | 10 | 0.03 |
| Negative | 43 | 21 | 22 |  |
| **T stage** |  |  |  |  |
| T1-T2 | 20 | 8 | 12 | 0.03 |
| T3 | 24 | 13 | 11 |  |
| T4 | 36 | 27 | 9 |  |
| **TNM stage** |  |  |  |  |
| I-II | 43 | 21 | 22 | 0.03 |
| III-IV | 37 | 27 | 10 |  |
| **Carcinoembryonic antigen** |  |  |  |  |
| Positive | 39 | 27 | 12 | 0.10 |
| Negative | 41 | 21 | 20 |  |
| **Carbohydrate antigen 199** |  |  |  |  |
| Positive | 26 | 13 | 13 | 0.20 |
| Negative | 54 | 35 | 19 |  |
